# Activation of recombinases at specific DNA loci by zinc-finger domain insertions

**DOI:** 10.1038/s41587-023-02121-y

**Published:** 2024-01-31

**Authors:** Liliya Mukhametzyanova, Lukas Theo Schmitt, Julia Torres-Rivera, Teresa Rojo-Romanos, Felix Lansing, Maciej Paszkowski-Rogacz, Heike Hollak, Melanie Brux, Martina Augsburg, Paul Martin Schneider, Frank Buchholz

**Affiliations:** 1 Medical Systems Biology, Medical Faculty, Technical University Dresden, Dresden, Germany; 2 Present Address: Seamless Therapeutics GmbH, Dresden, Germany

**Keywords:** Genetic engineering, Molecular evolution, Genetics research, Targeted gene repair

## Abstract

Recombinases have several potential advantages as genome editing tools compared to nucleases and other editing enzymes, but the process of engineering them to efficiently recombine predetermined DNA targets demands considerable investment of time and labor. Here we sought to harness zinc-finger DNA-binding domains (ZFDs) to program recombinase binding by developing fusions, in which ZFDs are inserted into recombinase coding sequences. By screening libraries of hybrid proteins, we optimized the insertion site, linker length, spacing and ZFD orientation and generated Cre-type recombinases that remain dormant unless the insertionally fused ZFD binds its target site placed in the vicinity of the recombinase binding site. The developed fusion improved targeted editing efficiencies of recombinases by four**-**fold and abolished measurable off-target activity in mammalian cells. The ZFD-dependent activity is transferable to a recombinase with relaxed specificity, providing the means for developing fully programmable recombinases. Our engineered recombinases provide improved genome editing tools with increased precision and efficiency.

## Abstract (web summary)

Recombinases fused with zinc-finger domains are active only when bound to their DNA targets.

## Main

Tyrosine site-specific recombinases (Y-SSRs), such as the Cre-loxP system, are widely used genome editing tools that hold potential for therapeutic application due to their precise mechanism of DNA manipulation. Y-SSRs can execute complex genome engineering operations, including excision, inversion, integration and cassette exchange of large genomic sequences, without inducing DNA double-stranded breaks and without relying on the cellular DNA repair machinery or additional co-factors. Therefore, the DNA editing process is predictable and works even in non-dividing cells^1^. However, laborious stepwise directed molecular evolution and protein engineering are required to reprogram them to target defined loci^2–12^. To address this limitation, recombinases were previously fused with exogenous DNA-binding domains^13–19^, but these approaches have not yet found widespread use, probably because of their low activity and/or because the recombinase domains are active independently of the DNA-binding domain, which could lead to off-target recombination. Ideally, recombination would occur only when the DNA-binding domain has recognized its intended DNA target site. Conditional recombinases were previously generated by fusion to ligand-binding domains of nuclear receptors^20–22^, rendering the enzymes dependent on binding of a ligand to the receptor domain. However, to our knowledge, recombinases whose activity depends on the binding of an exogenous domain to DNA have not yet been described.

In this study, we fused zinc-finger DNA-binding domains (ZFDs) to Cre-type recombinases. We performed systematic analyses to define the optimal spacing and orientation of the ZF-binding motif with respect to the recombinase recognition sequence as well as the optimal linker length between recombinase and ZFD. We found that N-terminal and C-terminal ZF–recombinase fusions increase recombination activity by up to 10-fold. We further applied pentapeptide scanning mutagenesis to identify positions that tolerate insertional fusions within the recombinase coding sequence. Notably, some insertional ZF–recombinase fusions render recombinase activity dependent on ZFD binding to its target sequence. We show that this approach improves the properties of a recombinase with therapeutic potential and establish a molecular evolution method, which allows to improve the properties of in silico designed ZFDs. Moreover, we developed a prototype of a multilateral recombinase that can be programmed to recombine many target sites in a ZF-dependent manner.

## Results

### N-terminal and C-terminal ZF fusions improve recombinase activity

**Fig. 1:**
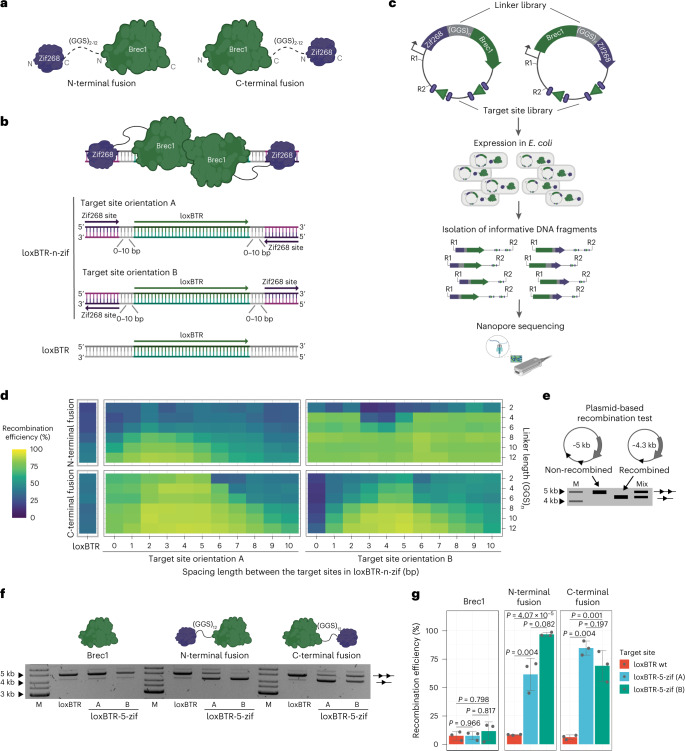
Optimization of the N-terminal and C-terminal Brec1–Zif268 fusions. **a**, Depiction of the fusion libraries, where the Zif268 DNA-binding domain is fused to the N-terminus or C-terminus of Brec1 recombinase with the flexible linker of 2, 4, 6, 8, 10 or 12 Gly-Gly-Ser repeats. **b**, Target site library overview. Important features are highlighted by colors and arrows. **c**, Schematic presentation of the plasmid recombination screen performed by nanopore sequencing. Important steps are highlighted by arrows. Recombinase and recombinase target sites (triangles) are shown in green. Zif268 and its target sites (ellipses) are shown in purple. Linkers are presented in gray. R1, restriction site 1; R2, restriction site 2. **d**, Heat map deduced from the nanopore sequencing results for N-terminal and C-terminal Brec1–Zif268 fusions. Recombination rates are mapped along the spacing length (*x* axis) and the linker length (*y* axis), respectively, for both target site orientations. The high-throughput screen was performed once. **e**, Schematic of the plasmid recombination assay. The difference between the recombined plasmids (line with one triangle) and the non-recombined plasmids (line with two triangles) can be detected by agarose gel electrophoresis. ‘Mix’ represents a mixture of the recombined and non-recombined plasmids, the ratio between which can be used to calculate the recombination efficiency. M, marker. **f**, Representative plasmid-based activity assay for indicated recombinase variants and target sites at low induction level (1 µg ml^−1^ ʟ-arabinose). Note the substantial increase in recombination activity for the Brec1–Zif268 fusions on the extended loxBTR targets (loxBTR-5-zif). **g**, Quantification of the recombination rates for indicated variants and target sites. Recombination efficiencies were calculated from ratios of recombined and non-recombined band intensities shown in **f**. The assay was performed three times (*n* = 3, biologically independent samples, plotted as dots). The bar graphs represent mean values, and the error bars indicate the standard deviation from the mean. Statistical relevance was assessed using an unpaired two-sided *t*-test. *P* values are indicated. Source data

To test whether fusions of a DNA-binding domain can improve properties of Cre-type engineered recombinases, we fused ZFDs to the N-terminus or C-terminus of these enzymes. We chose the ZFD of the EGR1 transcription factor Zif268 (refs. ^23,24^) and the Cre-derived recombinase Brec1 (ref. ^6^), because these proteins have been well characterized. Previous studies showed that different parameters in the designed fusion proteins, such as the linker length between the domains and the nucleotide distance between the target sites of DNA recognition components, can impact the activity of the fusion proteins^13,16,17^. Therefore, we created fusion libraries in which the linker length was varied by a different number of flexible GGS repeats (2–12) between Brec1 and Zif268 (Fig. 1a). In addition, we created a library of lox-zif target sites where the spacing between the Brec1 recognition site (loxBTR, 34 base pairs (bp)) and the 9-bp Zif268 binding motif varied from 0 bp to 10 bp. Moreover, we included two different orientations of the Zif268 binding sequences relative to the loxBTR half sites (Fig. 1b). To test all 276 possible combinations, we expressed the designed fusion complexes on the target site library from the pEVO plasmid^2^ in *Escherichia coli* and quantified the recombination efficiencies using nanopore sequencing by calculating the ratio of the recombined to the non-recombined plasmids (Fig. 1c).

The results revealed that optimal combinations of linker length, target site spacing and orientation displayed increased recombination rates compared to the non-fused recombinase, presumably because the ZFD binding to its site provided additional affinity to the DNA. The strongest improvements in activity for both N-terminal and C-terminal libraries were obtained with a linker of 12×GGS repeats and the spacer length between loxBTR and zif binding motif of 5 bp (Fig. 1d). Testing of the best combinations in a plasmid-based assay at low recombinase expression levels revealed on average 85% recombination efficiency (C-terminal Brec1–Zif268 fusion on loxBTR-5-zif (A) site), whereas WT–Brec1 recombined the plasmid on average to 7% under the same conditions (Fig. 1e–g). Hence, fusion of a ZFD markedly improved the recombination efficiency of an engineered recombinase.

**Extended Data Fig. 1:**
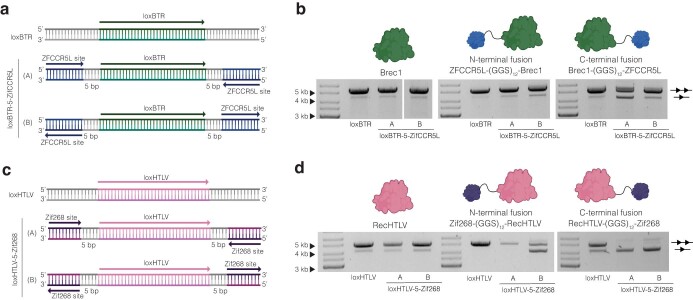
N- and C-terminal ZFD fusions of Brec1-ZFCCR5L and RecHTLV-Zif268 improve their activity. **a** Depiction of the target sites used for the activity assay of Brec1-ZFCCR5L. Recombination efficiency was tested on two types of target sites: recombinase loxBTR (34 bp) target site only, or the loxBTR-5-zifCCR5L target sites where the loxBTR is flanked by the ZFCCR5L binding motif (12 bp) in two different orientations (A and B) relative to each other and the distance of 5 bp between the binding sites. **b** Plasmid-based activity assay for Brec1-ZFCCR5L fusions on loxBTR, loxBTR-5-zifCCR5L (A), or loxBTR-5-zifCCR5L (B). The test was performed at 1 ug/ml L-arabinose. Activity of the wild-type Brec1 is shown as a control. **c** Depiction of the target sites used for the activity assay of RecHTLV-Zif268. Recombination efficiency was tested on two types of target sites: recombinase loxHTLV (34 bp) target site only, or loxHTLV-5-zif target sites where the loxHTLV is flanked by Zif268 binding motives (9 bp) in two different orientations (A and B) relative to each other and the distance of 5 bp between the binding sites. **d** Plasmid-based activity assay for RecHTLV-Zif268 fusions on loxHTLV, loxHTLV-5-zif268 (A), or loxHTLV-5-zif268 (B). The test was performed at 10 ug/ml L-arabinose. Activity of the wild-type RecHTLV is shown as a control. For (**b**) and (**d**) the upper band represents the unrecombined plasmid (line with two triangles), and the lower band represents the recombined plasmid (line with one triangle). M = Marker. The assays were performed three times (n = 3 biologically independent replicates).

To test the versatility of the developed fusion architectures, we performed ZF–recombinase fusions with an engineered ZFD (ZFCCR5L (ref. ^25^)) as well as with another Cre-derived recombinase (RecHTLV (ref. ^9^)). Both Brec1–ZFCCR5L and RecHTLV–Zif268 fusions exhibited similar improvements in recombination activity as seen for Brec1–Zif268 (Extended Data Fig. 1), indicating that the obtained results can be transferred to other engineered recombinases and ZFDs. We conclude that optimized combinations of linkers, target site spacing and orientation can be used for fusion of ZFDs on the N-termini and C-termini of evolved Cre-type recombinases to improve their activity on lox-zif target sites.

### Pentapeptide scanning mutagenesis of Cre-type recombinases

**Fig. 2:**
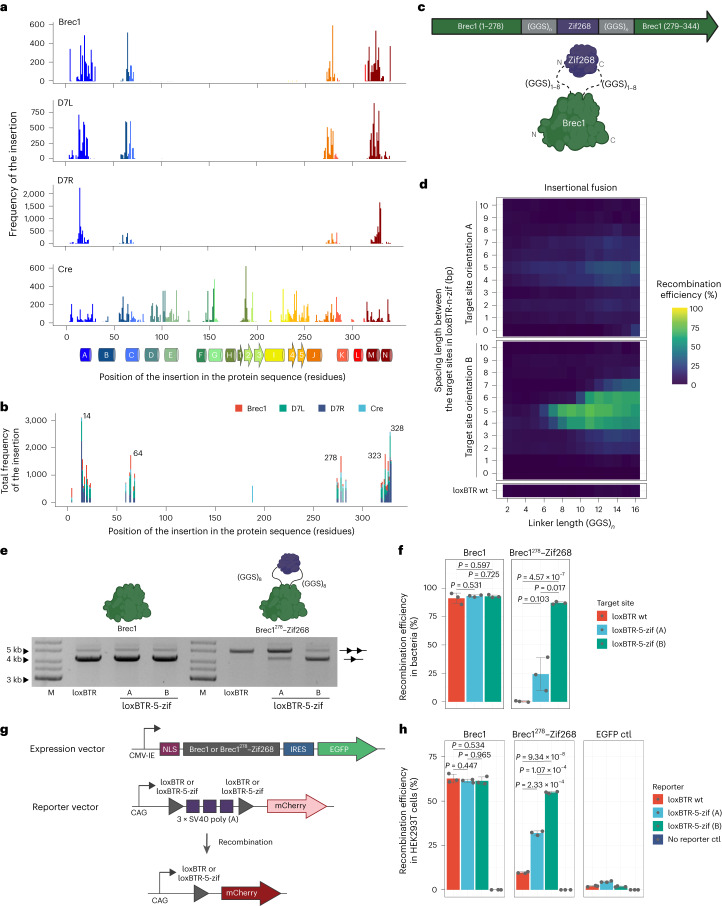
Insertional ZFD fusions generate ZF-dependent recombinase systems. **a**, Results from the pentapeptide scanning mutagenesis screens. Frequencies and distribution of the five-amino-acid insertions in the sequences of active recombinases are shown. Secondary structure elements are indicated: alpha-helices are displayed as cylinders with letters, and beta-sheets are represented as numbered arrows. The residues are color-coded according to the secondary structure of Cre (ref. ^1^). **b**, Cumulative frequencies of insertions for Brec1 (red), D7L (green), D7R (dark blue) and Cre (light blue). The top five residues with the highest insertion frequencies are indicated with their amino acid positions. **c**, Depiction of the insertional fusion library, where the Zif268 DNA-binding domain (purple) is flanked with flexible linkers of 1–8 Gly-Gly-Ser repeats (gray), inserted into the Brec1 recombinase sequence (green) between residues 278 and 279. **d**, Heat map deduced from the nanopore sequencing results for the insertional Brec1–Zif268 fusions. For simplicity of the visualization, the linker length is shown as a sum of Gly-Gly-Ser repeats in the left and right linker. **e**, Representative plasmid-based activity assay for indicated recombinase variants and target sites at high induction level (200 µg ml^−1^ ʟ-arabinose). The upper band represents the unrecombined plasmid (line with two triangles). The lower band represents the recombined plasmid (line with one triangle). M, marker. **f**, Quantification of recombination rates from band intensities shown as in **e**. **g**, Schematic representation of the plasmid constructs used for the recombination assay in HEK293T cells. Important elements of the expression and reporter plasmids are indicated. Upon recombination of the target sites on the reporter, the 3×SV40poly(A) is excised, allowing for the expression of the red fluorescent protein (mCherry). **h**, Quantification of recombination efficiencies in HEK293T cells 48 h after transfection for indicated variants, analyzed by flow cytometry. Recombination rates were calculated as the percentage of the recombined cells (mCherry positive) normalized for transfection efficiency (GFP positive). For **f** and **h**, the assay was performed three times (*n* = 3, biologically independent samples, plotted as dots). The bar graphs represent mean values, and the error bars indicate the standard deviation from the mean. Statistical relevance was assessed using a two-sample *t*-test. *P* values are indicated. ctrl, control. Source data

Another possible approach for generating ZF–recombinase fusions involves inserting the ZFD sequence into the coding sequence of the recombinase. However, such insertional fusions could potentially render the enzyme non-functional. To identify positions within engineered recombinases that can tolerate small insertions, we performed pentapeptide scanning mutagenesis^26,27^ using four different recombinases, namely Cre, Brec1 (ref. ^6^), D7L and D7R (ref. ^8^). Using in vitro Mu transposition, we created four libraries where five-amino-acid in-frame insertions were introduced throughout the reading frames of the recombinases and selected the variants that retained recombinase activity, followed by long-read sequencing (Supplementary Fig. 1). Mapping the reads revealed that insertions in Cre were tolerated at numerous positions, reflecting the robustness of the enzyme for insertional mutagenesis^27^ (Fig. 2a). In contrast, the Cre-derived recombinases did not exhibit the same pattern, with fewer regions allowing insertions of the five amino acids. However, the active mutants of Cre, Brec1, D7L and D7R contained insertions in identical regions of the proteins, suggesting these areas as potential universal insertion sites (Fig. 2b). Based on the frequency as well as the spatial accessibility and proximity of the residues to the DNA (Supplementary Fig. 2 and Supplementary Note 1), the position between residues 278 and 279 was selected for further analyses.

### Insertional ZFD fusions yield ZF-dependent recombinases

Following the example of the terminal fusions, we used a high-throughput screen to develop the architecture for the insertional fusions with ZFDs. We created a library by fusing Zif268 between residues 278 and 279 in Brec1 using two linkers, each consisting of 1–8 GGS repeats (Fig. 2c), and subsequently expressed this library alongside the previously developed library of target sites (Fig. 1b). The resulting 1,472 combinations were tested using nanopore sequencing technology (Fig. 1c). Despite the compatibility of the five-amino-acid insertion, all of the variants carrying the insertional Zif268 fusion were inactive on wt loxBTR, even at high recombinase expression level, indicating that the insertion of Zif268 between residues G278 and S279 disrupted the activity of Brec1 (Fig. 2d and Supplementary Fig. 3). In sharp contrast, optimal combinations of the linkers, target site orientation and spacing lengths unmasked recombination activity on some of the loxBTR-n-zif target sites, implying that binding of the ZFDs to their target site was required to recover recombination activity. Overall, a strong preference for longer linkers, 5-bp spacing and the target site orientation B was observed. We tested the best variant where Zif268 was fused with 8×GGS linkers, hereafter referred to as Brec1^278^–Zif268, in a plasmid-based assay (schematic shown in Fig. 1e). Consistent with the screen results, the Brec1^278^–Zif268 fusion showed impaired activity on the wt loxBTR, even at high induction level of 200 µg ml^−1^ ʟ-arabinose, but regained full activity on the loxBTR-5-zif (B) sites (Fig. 2e,f). Therefore, the insertional ZFD fusion generated a recombinase that is dependent on binding of the ZFD to its target sequence for recombination activity.

To investigate functionality of the ZF–recombinase fusion in human cells, we transiently co-transfected Brec1 or Brec1^278^–Zif268 expression constructs together with fluorescent recombination-reporter plasmids into HEK293T cells (Fig. 2g). As expected, Brec1 did not distinguish between the loxBTR and loxBTR-5-zif target sites and recombined both reporter plasmids at a rate of around 60%. The activity of Brec1^278^–Zif268 in HEK293T cells was severely impaired on the wt loxBTR plasmid (although not as pronounced as in *E. coli*), whereas almost full recombination activity was observed on the loxBTR-5-zif (B) (Fig. 2h and Supplementary Fig. 4). Overall, these results indicate that the insertional ZF–recombinase fusion architecture generates ZF-dependent recombination systems in human cells.

**Extended Data Fig. 2:**
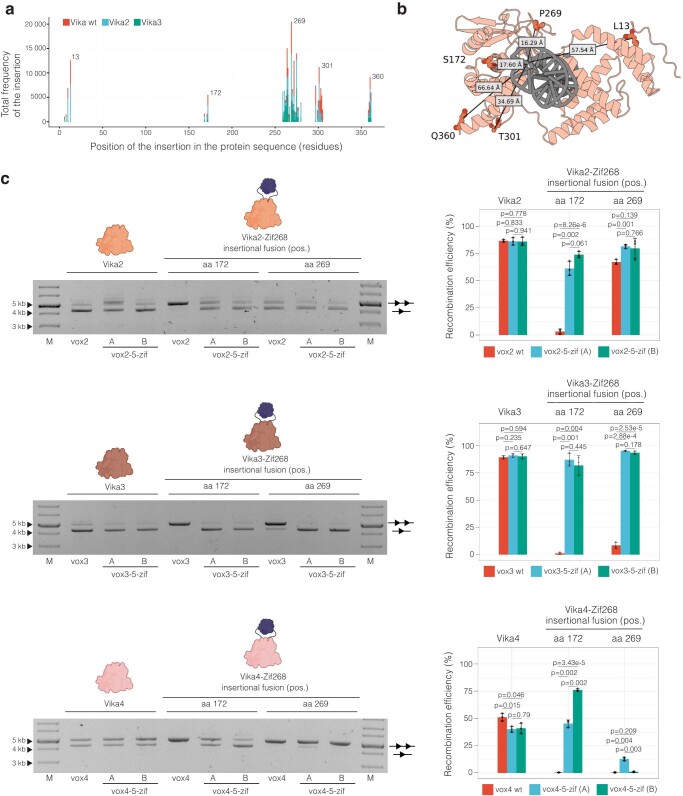
Application of the developed pipeline to obtain ZF-dependent Vika-type Y-SSRs. **a** Pentapeptide scanning mutagenesis and selection of the active Vika-type recombinase variants. Cumulative frequencies of insertions for active variants are shown and the top 5 insertion positions indicated. **b** Prediction and analysis of the 3D protein structure of Vika. The 3D model of Vika wt was predicted using AlphaFold^67^ and superimposed with the monomer of the Cre/loxP synapse (PDB ID 1Q3U^68^). The most frequent positions that tolerated insertions are highlighted in dark orange and the amino acid is indicated. The distances between the selected residues and the DNA are indicated. The image was created using the 3D Protein imager^69^. **c** Plasmid-based activity assay for created fusions, in which the Zif268 was fused between the residues 172 and 173 or 269 and 270 (using (GGS)_8_ linkers) of Vika2, Vika3, and Vika4 (Vika4 was not part of the pentapeptide scanning mutagenesis). The activities on the respective recombinase target sites (vox2, vox3, vox4) and their vox-5-zif (A) and (B) versions were tested. The test was performed at 100 ug/ml L-arabinose. Activity of the wild-type recombinases is shown as a control. The upper band represents the unrecombined plasmid (line with two triangles), the lower band represents the recombined plasmid (line with one triangle). M = Marker. On the right side the quantification of the recombination efficiencies were calculated from ratios of recombined and non-recombined band intensities. The assay was performed three times (n = 3 biologically independent replicates, plotted as dots), the bar graph represents the mean value, the error bars indicate the standard deviation from the mean. Statistical relevance of the triplicates was assessed using an unpaired two-sided t-test. P-values are indicated. Some recombination on the vox-like sites is observed for the complexes, when the fusion between the position aa269 and 270, indicating the lower stringency of this position. However, the fusion complexes with the ZFD insertion between the residues 172 and 173 resemble the ZF-dependent phenotype observed for Cre-like recombinases. Therefore, identifying the most suitable position for ZFD fusion is crucial for generation of ZF-coupled recombinases.

To investigate whether the approach can be applied to another member of tyrosine recombinases, we performed pentapeptide scanning mutagenesis screens and tested fusions of Zif268 with Vika, a recombinase isolated from *Vibrio coralliilyticus*^28^, and three evolved Vika-based recombinases. Notably, we were able to replicate results obtained with the Cre-type enzymes, demonstrating the portability of the approach (Extended Data Fig. 2 and Supplementary Note 1).

**Extended Data Fig. 3:**
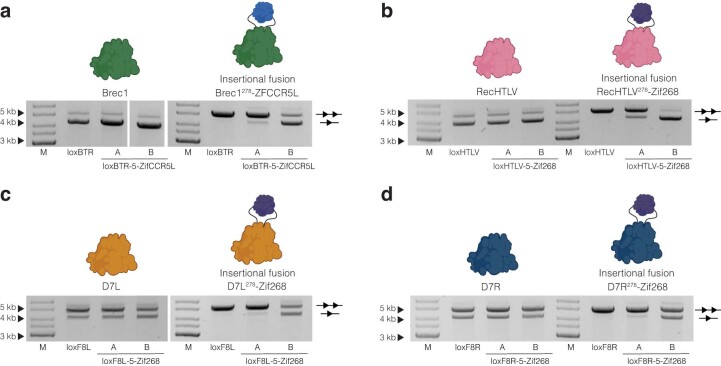
Activity tests of ZF-dependent designer-recombinases. **a** Plasmid-based activity assay for Brec1^278^-ZFCCR5L, in which the ZifCCR5L is fused between the residues 278 and 279 of Brec1 via the (GGS)_8_ linkers. The fusion complex was tested on loxBTR, loxBTR-5-zifCCR5L (A), or loxBTR-5-zifCCR5L (B) target sites. The test was performed at 200 ug/ml L-arabinose. Activity of the wild-type Brec1 is shown as a control. **b** Plasmid-based activity assay of RecHTLV^278^-Zif268 fusion complex, in which the Zif268 is fused between the residues 278 and 279 of RecHTLV via the (GGS)_8_ linkers. The fusion complex was tested on loxHTLV, loxHTLV-5-zif268 (A), or loxHTLV-5-zif268 (B) target sites. The test was performed at 100 ug/ml L-arabinose. Activity of the wild-type RecHTLV is shown as a control. **c** Plasmid-based activity assay for D7L^278^-Zif268, in which the Zif268 is fused between the residues 278 and 279 of D7L via the (GGS)_8_ linkers. The fusion complex was tested on loxF8L, loxF8L-5-zif268 (A), or loxF8L-5-zif268 (B) target sites. The test was performed at 10 ug/ml L-arabinose. Activity of the wild-type D7L is shown as a control. **d** Plasmid-based activity assay for D7R^278^-Zif268, in which the Zif268 is fused between the residues 278 and 279 of D7R via the (GGS)_8_ linkers. The fusion complex was tested on loxF8R, loxF8R-5-zif268 (A), or loxF8R-5-zif268 (B) target sites. The test was performed at 200 ug/ml L-arabinose. Activity of the wild-type D7R is shown as a control. For (**a**), (**b**), (**c**) and (**d**) the upper band represents the unrecombined plasmid (line with two triangles), and the lower band represents the recombined plasmid (line with one triangle). M = Marker. The assays were performed three times (n = 3 biologically independent replicates).

To explore the versatility of the approach with other ZFDs and other engineered recombinases, we tested insertional fusion of Brec1 with ZFCCR5L (ref. ^25^) that carries an additional zinc-finger and recognizes a 12-bp sequence and Zif268 fusion with D7L, D7R (ref. ^8^) and RecHTLV (ref. ^9^). All fusion complexes exhibited the ZF-dependent phenotype observed for Brec1^278^–Zif268 (Extended Data Fig. 3). RecHTLV was not included in the pentapeptide scanning mutagenesis screen, suggesting that ZFD insertions between residues 278 and 279 consistently generate Cre-type engineered recombinases that are activated by ZF DNA binding. We conclude that insertional ZFD fusions can create ZF-dependent Cre-type recombinases, providing the means for generating highly active and specific engineered recombinases.

### Design and directed evolution of ZFDs for a genomic locus

**Fig. 3:**
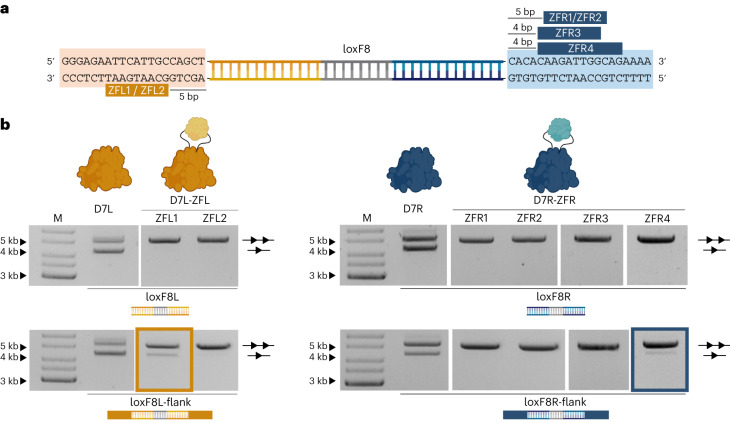
Design of ZFDs for the loxF8 genomic locus. **a**, Genomic sequences flanking loxF8. Binding sites of the designed ZFDs upstream (orange, ZFL1–2) and downstream (blue, ZFR1–4) are shown with the distance (bp) between the loxF8 and the ZF motifs indicated. **b**, Representative plasmid-based activity assay of the monomers from the D7 recombinase heterodimer (D7L and D7R) fused with the designed ZFDs on the symmetric loxF8L and loxF8R target sites and their extended versions that include the respective flanking genomic sequences for ZFD binding (loxF8L-flank and loxF8R-flank). The activity of the wild-type monomers is shown as a control. High induction levels (100 µg ml^−1^ ʟ-arabinose for D7L and D7L–ZFL; 200 µg ml^−1^ ʟ-arabinose for D7R and D7R–ZFR) were used. The upper band represents the unrecombined plasmid (line with two triangles), and the lower band represents the recombined plasmid (line with one triangle). The assay was performed three times (*n* = 3, biologically independent samples; replicates are shown in Source Data files). M, marker. Fusions with activity (D7L–ZFL1 and D7R–ZFR4) are highlighted with an orange box and a blue box, respectively. Source data

**Fig. 4:**
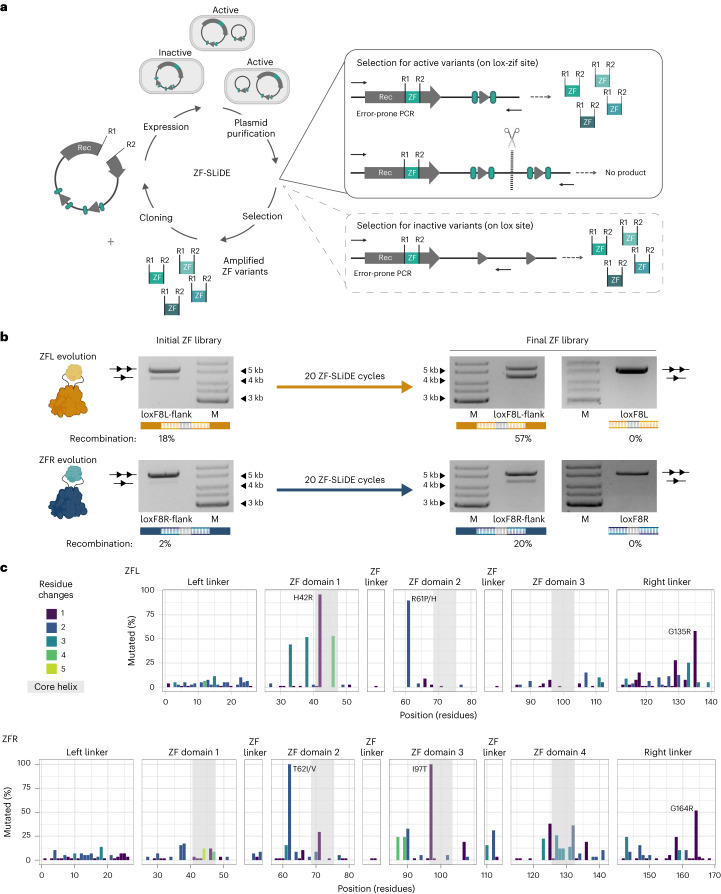
Substrate-linked directed evolution of zinc-finger domains. **a**, Schematic presentation of substrate-linked directed molecular evolution for zinc-finger domains (ZF-SLiDE). Evolution cycles start with cloning the ZFD library between residues 278 and 279 of the recombinase sequence encoded in the pEVO vector. Additionally, the vector carries two lox-zif target sites of interest (lox sites are indicated as triangles; zif motifs are indicated as ellipses flanking the lox sites). After expression of the ZFD–recombinase fusion, plasmid DNA is isolated and analyzed. Upon successful recombination, a unique restriction site (indicated as scissors) between two target sites is excised. By applying restriction digestion, the non-recombined plasmids are linearized, whereas the recombined plasmids remain circular. The digestion is followed by a PCR using indicated primers (arrows), which generates product only from recombined plasmids. Successful ZF variants are then subjected to the next round of directed evolution. Counter-selection is applied with vectors containing the lox sites of interest alone, without the zif motifs. **b**, ZF-SLiDE progress assessed by plasmid-based recombination assay. Recombination efficiency of D7L and D7R fused with the ZF libraries on the loxF8Lflank and loxF8Rflank target sites, respectively, is shown at the start of directed evolution and after the 20 cycles of ZF-SLiDE. Activities of the final libraries on the loxF8L and loxF8R sites are shown as a control. All tests were performed at high induction level (200 µg ml^−1^ ʟ-arabinose). Recombination efficiencies were calculated from band intensities and are indicated in percent underneath the gel pictures. The evolution and the recombination assay of the libraries were performed as a single experiment (*n* = 1). M, marker. **c**, Sequence analysis of the evolved ZFDs obtained by sequencing 78 and 59 active variants from the final ZFL and ZFR libraries, respectively. The frequency of the mutated residues compared to the designed ZFD that served as a starting point (ZFL1 and ZFR4) is shown. The number of different amino acids identified at a particular position is color-coded (residue changes). The core helices of the ZFDs are highlighted in gray. The most conserved mutations are indicated by the residue number and the amino acid change. Source data

To test the developed approach on a natural human genomic locus, we chose to apply it to the heterodimeric Cre-derived recombinase D7, recently developed for correcting the 140-kb genomic int1h inversion causing hemophilia A (ref. ^8^). We designed ZFL1 and ZFL2 for the human genomic sequence upstream of the loxF8 target sites in the F8 gene and ZFR1–4 for the downstream sequence, using publicly available platforms^29,30^ (Fig. 3a and Supplementary Table 1). We tested the activity of the monomers (D7L and D7R) fused at position 278 with the designed ZFDs on the respective symmetric sites (loxF8L and loxF8R) and their extended versions that included 20 bp of the genomic flanking sequences (loxF8L-flank and loxF8R-flank) (Fig. 3b). Consistent with previous results, none of the tested complexes showed activity on loxF8L and loxF8R. In contrast, two of the fusions (D7L–ZFL1 and D7R–ZFR4) showed activity on the extended target sites, albeit at a lower efficiency when compared to the non-fused recombinases. To improve the designed ZFDs, we adopted the well-established substrate-linked directed evolution (SLiDE) protocol^2^ for the directed evolution of ZFDs. We created libraries of randomly mutated ZFL1 and ZFR4 and cloned these libraries into a modified evolution vector, containing the recombinase sequence and the respective target sites (Fig. 4a). We performed 20 cycles of mutagenesis, selection and counter-selection to improve specific ZFD binding to the flanking sites and observed a substantial increase in recombination activity of the final libraries on the extended target sites, indicating that ZFDs with improved properties had evolved (Fig. 4b).

**Extended Data Fig. 4:**
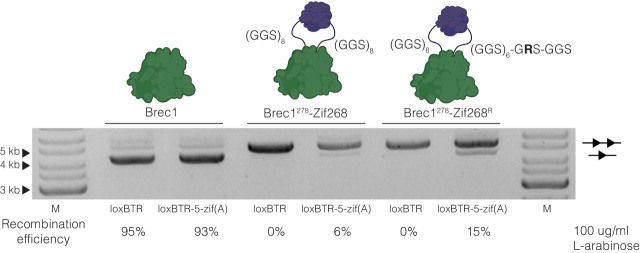
A mutation in the linker improves recombination efficiency of Brec1^278^-Zif268. Plasmid-based activity assay for Brec1^278^-Zif268 containing a mutation in the right linker (Brec1-(GGS)_8_-Zif268-(GGS)_6_-G**R**S-GGS) compared to Brec1^278^-Zif268 (Brec1-(GGS)_8_-Zif268-(GGS)_8_) on the loxBTR and loxBTR-5-zif (A) target sites. The test was performed at 100 ug/ml L-arabinose. Activity of the wild-type Brec1 is shown as a control. Recombination efficiencies were calculated from ratios of recombined and non-recombined band intensities. The assays were performed three times (n = 3 biologically independent replicates). The upper band represents the unrecombined plasmid (line with two triangles), and the lower band represents the recombined plasmid (line with one triangle). M = Marker.

Sequence analyses of active clones in the final ZF libraries uncovered conserved acquired mutations (Fig. 4c and Supplementary Figs. 5 and 6). Some of these mutations were observed in the core helices of domain 1 in the ZFL library and domain 3 in the ZFR library, suggesting that these residues are crucial for the improved properties of the evolved ZFDs. We also observed conserved mutations in the scaffold of the ZFDs, consistent with a recent report that scaffold optimization helps to improve ZFDs^31^. Furthermore, a conserved G-to-R mutation was observed in the right linker of both ZF libraries. To test whether this mutation alone contributed to the improved properties of the fusions, we introduced it into the right linker of the Brec1^278^–Zif268 complex and indeed observed an increased recombination efficiency (2.5-fold) on the loxBTR-5-zif (A) site (Extended Data Fig. 4). Altogether, we established a straightforward pipeline to improve the properties of ZFDs for downstream applications.

### D7-ZF exhibits improved applied properties

**Fig. 5:**
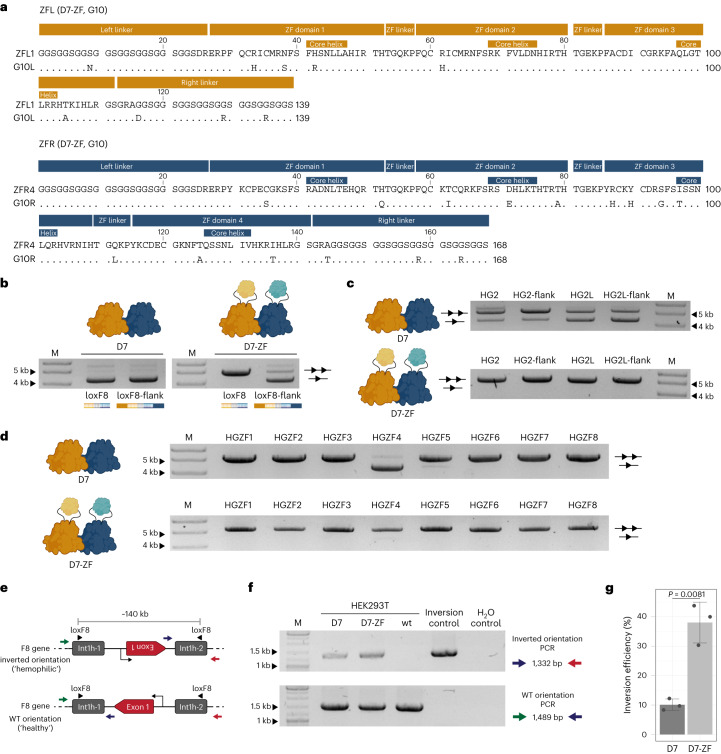
Characterization of D7-ZF. **a**, Mutation analysis of D7-ZF (G10-ZFL and G10-ZFR) (one-letter code). The amino acid sequence of ZFL1 and ZFR4 and the (GGS)_8_ linkers are shown as a reference. Dots indicate conserved residues. **b**, Representative plasmid-based activity assay of D7-ZF on the loxF8 and loxF8-flank that includes the flanking genomic sequences for the ZFD binding target sites as found in the human genome. **c**, Representative plasmid-based activity assay of D7-ZF on predicted human genomic loxF8-like off-targets that are recombined by D7 and their extended versions that include the flanking genomic sequences upstream and downstream of the lox sites. **d**, Representative plasmid-based activity assay of D7 and D7-ZF on predicted human genomic loxF8-like off-targets flanked with the sequences potentially recognized by D7-ZF. For **b**–**d**, tests were performed at high induction level (100 µg ml^−1^ ʟ-arabinose). The upper band represents the unrecombined plasmid (line with two triangles); the lower band represents the recombined plasmid (line with one triangle). M, marker. Activity of D7 is shown as a control. The assays were performed three times (*n* = 3, biologically independent samples; replicates are shown in Source Data files). **e**, Schematic representation of a fraction of the F8 gene, displaying the wild-type and inverted orientations of the loxF8 locus (adapted from ref. ^8^), with colored arrows indicating primers used for PCR to detect the orientation. Black arrow indicates the transcription start site of the F8 gene. **f**, Agarose gel image of PCR carried to detect the orientation of the loxF8 locus in the F8 gene of the HEK293T cells 72 h after transfection with D7 or D7-ZF. The non-treated HEK293T cells were used as a wild-type control. The iPSCs derived from a patient carrying the Exon1 inversion were used as an inversion control. M, marker. **g**, Inversion efficiencies of the loxF8 locus in HEK293T cells 72 h after transfection with D7 and D7-ZF, quantified by qPCR. The assay was performed three times (*n* = 3, biologically independent samples, plotted as dots). The bar graphs represent mean values, and the error bars indicate the standard deviation from the mean. Statistical relevance was assessed using an unpaired two-sided *t*-test. *P* values are indicated. Source data

**Extended Data Fig. 5:**
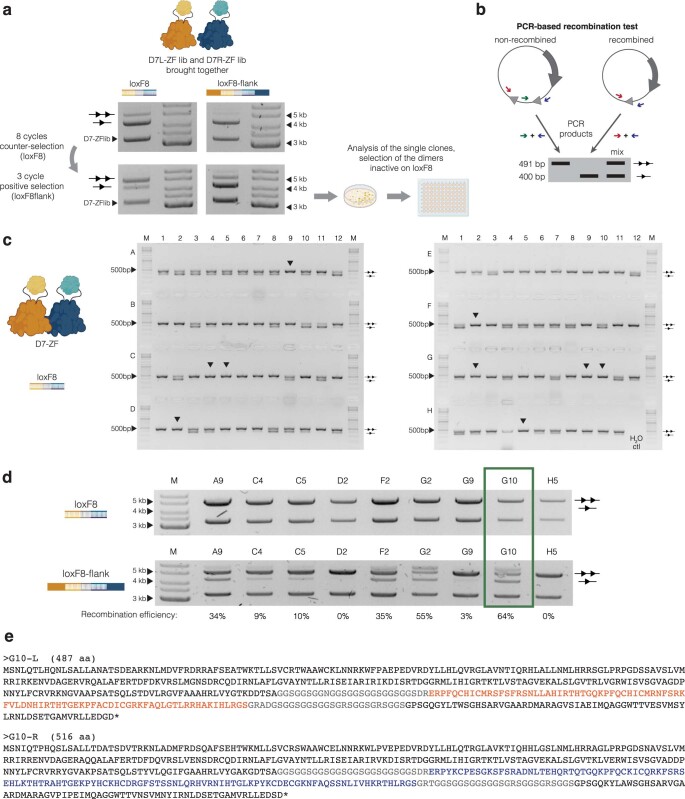
Identification of D7-ZF. **a** Overview of the heterodimer positive and counter-selection and the clone selection procedure. Plasmid-based activity assays are shown for the D7 recombinase containing the ZFL and ZFR libraries on the loxF8 and loxF8-flank target sites. Activities of the initial and the final library after 8 cycles of counter-selection on loxF8 and 3 cycles of positive selection on loxF8-flank are shown. 100 ug/ml L-arabinose was used for the initial library on loxF8-flank, 200 ug/ml L-arabinose for the initial library on loxF8 and for the final library on loxF8 and loxF8-flank. After the selection, single clones were picked and their activity was analysed by PCR. **b** Schematic representation of the PCR-based recombination test used for single clone analyses (adapted from Lansing et al.^8^). Primers are indicated as coloured arrows. Three-primer PCR generates a bigger fragment (491 bp) from the non-recombined pEVO plasmid, or a smaller fragment (400 bp) from the recombined plasmid, if a mix of both plasmids used for the PCR both bands are detected. **c** 95 heterodimer D7-ZF clones were expressed on loxF8 target site in *E.coli* and analysed by the PCR-based recombination test (n = 1). Clones that did not recombine the loxF8 target site and were selected for further analysis and are marked with black triangles. **d** Plasmid-based activity assay for the candidate D7-ZF clones on the loxF8 and loxF8-flank. 200 ug/ml L-arabinose was used for the test on loxF8 and 10 ug/ml L-arabinose on loxF8-flank. Recombination efficiencies were calculated from ratios of recombined and non-recombined band intensities. Activity of the most efficient clone (D7-ZF, G10) that was selected for future experiments is highlighted by a green box. The assay was performed three times (n = 3 biologically independent replicates). For (**a-d**), the upper band represents the unrecombined plasmid (line with two triangles), the lower band represents the recombined plasmid (line with one triangle). M = Marker. **e** Amino acid sequence of the selected monomers of the D7-ZF (G10, one-letter code). The sequence of the ZFL is highlighted in orange, the sequence of the ZFR is highlighted in blue, the sequence of the linkers is highlighted in grey. Asterisks denote stop codons.

We next combined the two monomer libraries and performed several rounds of selection for activity of the recombinase heterodimer fused with the ZFDs on the final extended loxF8 target site as it is found in the human genome (Extended Data Fig. 5a). We selected clone G10 (hereafter referred to as D7-ZF) for further studies, because it showed high recombination activity on loxF8-flank (64% at 10 µg ml^−1^ ʟ-arabinose) and no activity on loxF8 (at 200 µg ml^−1^ ʟ-arabinose) (Fig. 5a,b and Extended Data Fig. 5b–e). Sequence analysis revealed that, during directed evolution, the ZFL and ZFR acquired five and 12 mutations, respectively, as well as four and three mutations in the linkers, including two conserved G-to-R changes in their right linkers, which likely contributed to their advantageous properties (Fig. 5a).

**Extended Data Fig. 6:**
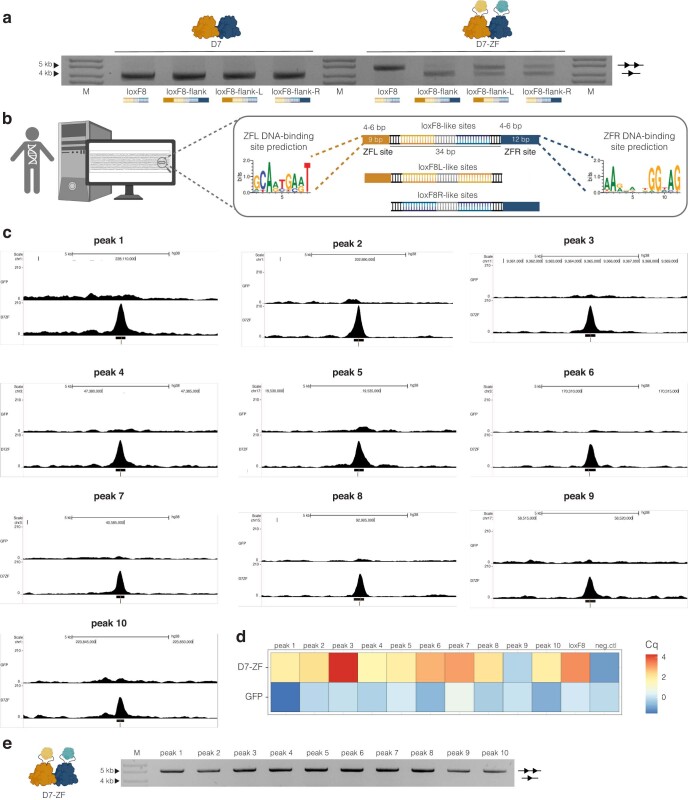
D7-ZF off-target analysis. **a** Plasmid-based activity assay of D7-ZF on loxF8 and its extended versions. The extended sequences for the flanking genomic sequence for the ZFL from the left site (loxF8-flank-L) or the flanking genomic sequence for the ZFR from the right site (loxF8-flank-R) are indicated and color-coded. Activity of the wild-type D7 recombinase heterodimer is shown as a control. The test was performed at high induction level (100 ug/ml L-arabinose). The assay was performed three times (n = 3 biologically independent replicates). The upper band represents the unrecombined plasmid (line with two triangles), and the lower band represents the recombined plasmid (line with one triangle); M = Marker. **b** Schematic depiction of the bioinformatic loxF8-ZF off-target search. The previously predicted loxF8-like target sites^8^ were screened for the presence of both or one of the two potential ZFD DNA binding sites (position weight matrices of the predicted sites are shown on the left side for the ZFL and on the right side for the ZFR). **c** Unbiased experimental identification of putative human off-target sites by ChIP-Seq. ChIP-seq pileups at the loci of the selected peaks for the D7-ZF sample and GFP control are shown. Chromosomal positions and a size bar are indicated for each plot. **d** qPCR-based validation of putative D7-ZF binding sites identified by ChIP-seq. The GFP sample was used as a control. The heatmap displays the enrichment as a difference of the Cq in the input sample and in the IP sample for each tested peak. **e** Plasmid-based bacterial D7-ZF activity assay on the ten identified D7-ZF binding sites. M = Marker. The assay was performed three times (n = 3 biologically independent replicates). Source data

**Extended Data Fig. 7:**
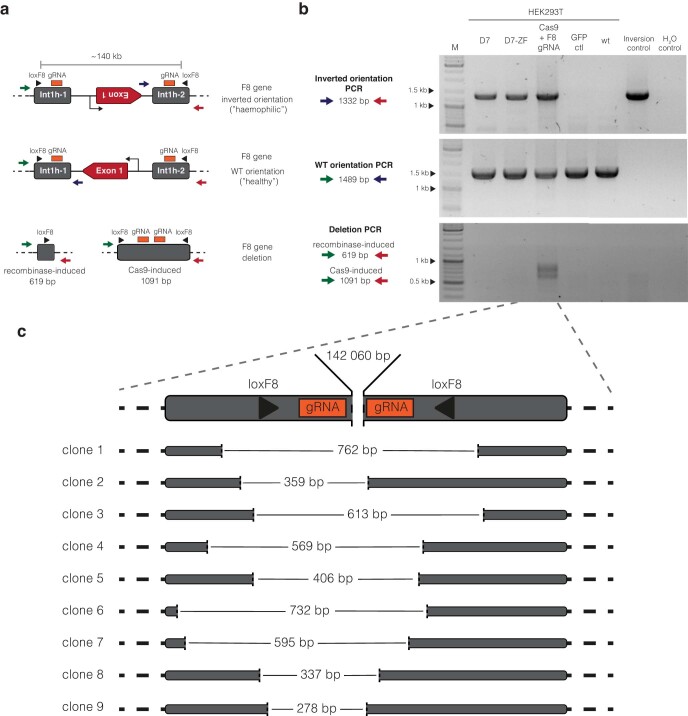
Comparison of recombinase and CRISPR/Cas9 approaches for the presence of unintended deletion on the endogenous F8 locus in human cells. Detection of inversion and excision (deletion) on the F8 locus in HEK293T cells assessed 48 h post mRNA transfection with D7, D7-ZF or Cas9 in combination with a gRNAs specific for the inverted repeat as published by Park et al.^32^. **a** Schematic representations of a fraction of the F8 gene, displaying the two possible orientations of the loxF8 locus (wild-type and inverted orientation) and two possible deletion outcomes (recombinase- or Cas9-induced) are shown (adapted from Lansing et al.^8^). Primers used for PCR to detect the orientation of the locus are displayed as green, blue, and red arrows. The position of the loxF8 sites, gRNA binding sites and the distance between them are indicated. **b** Agarose gel images of PCR products generated using the indicated primer combinations. The HEK293T cells treated with GFP mRNA only were used as a wild-type control, whereas iPSCs derived DNA from a patient carrying the int1h inversion were used as an inversion control. Primer combinations are indicated. M = Marker. The assay was performed three times (n = 3 biologically independent replicates). **c** Depiction of a potential precise Cas9-induced 142 060 bp deletion between the two gRNA binding sites. Sequences of the clones obtained from TA cloning of the deletion product induced by Cas9 are schematically shown below, indicating the size of the genomic fragments that were deleted in addition to the 142 060 bp.

To further investigate possible improvements of D7-ZF, we tested it on human genomic off-targets HG2 and HG2L that were previously reported to be recombined by the D7 heterodimer^8^. In contrast to D7, no activity was observed for D7-ZF on these off-targets or on their extended versions, which included the genomic sequences upstream and downstream of the 34-bp pseudo-loxF8 sites (Fig. 5c). To evaluate whether D7-ZF shows activity if only one of the flanking genomic sequences for the ZFD binding sites is present adjacent to the recombinase lox site, we tested the heterodimer on loxF8-flank-L and loxF8-flank-R target sites. Presence of only one of the ZFD binding sites substantially reduced recombination activity of D7-ZF, but it did not fully abolish it (Extended Data Fig. 6a). To test whether D7-ZF possibly gained activity on new off-targets due to the presence of the additional DNA-binding domains, we bioinformatically screened the human genome for lox sites with flanking sequences potentially recognized by the evolved ZFDs from one or both sides of the lox sites (Extended Data Fig. 6b and Supplementary Table 2). We tested D7 and D7-ZF on eight of the identified potential human off-targets (HGZF1–8). D7 recombined two of these off-targets (HGZF4 and HGZF5), whereas no recombination activity was detected for D7-ZF on any of the sites, demonstrating its high specificity and suggesting that this approach does not lead to new off-targets (Fig. 5d). To identify potential human genomic off-targets in an unbiased experimental approach, we performed a chromatin immunoprecipitation followed by sequencing (ChIP-seq)-based assay^8,9^. Twenty-five high-confidence D7-ZF putative binding sites were found using this method (Supplementary Table 3). The top 10 hits were selected for validation and recombination activity tests with a highly sensitive plasmid assay in *E. coli* (Extended Data Fig. 6c–e). D7-ZF did not show any recombination activity on any of these sites (Extended Data Fig. 6e), indicating that, although these sequences are bound, no recombination by D7-ZF is triggered at these sites. We also explored the potential unintended deletion of the 140-kb fragment and conducted a comparative analysis between the recombinase approach and Cas9 nuclease^32^. As anticipated, our PCR analysis revealed the presence of fragments indicative of substantial deletions in cells subjected to Cas9 nuclease treatment (Extended Data Fig. 7a–c). Conversely, in samples treated with D7 or D7-ZF, no bands specific to deletions were observed (Extended Data Fig. 7a,b), documenting the lack of unwanted excision events using the recombinase approach. Altogether, these results show that insertional ZFD fusions within an engineered recombinase with therapeutic potential can improve its applied properties.

**Extended Data Fig. 8:**
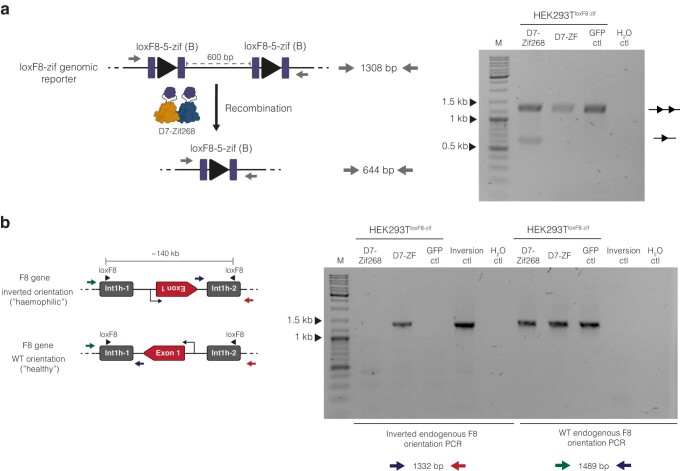
Recombination activity of D7^278^-Zif268 in human cells. **a** Recombination activity of D7^278^-Zif268 and D7-ZF on the genomic loxF8-zif reporter, carrying the Zif268 motifs flanking the loxF8 target site in HEK293T^loxF8-zif^ cells two days after mRNA transfections. Schematic representation of the unrecombined and recombined genomic loxF8-zif reporter is shown to the left. Primers used for PCR to detect the recombination of the reporter are depicted in grey. The distance between the loxF8-5-zif(B) target sites and the size of the possible PCR products is indicated. An agarose gel image of PCR products generated using the indicated primers to detect reporter recombination is shown to the right. The HEK293T^loxF8-zif^ cells treated with GFP mRNA only were used as a wild-type control. The upper band represents the unrecombined product (line with two triangles), and the lower band represents the recombined product (line with one triangle); M = Marker. **b** Inversion efficiency of D7^278^-Zif268 and D7-ZF on the endogenous loxF8 locus in HEK293T^loxF8-zif^ cells two days post mRNA transfection. Schematic representation of a fraction of the F8 gene, displaying the two possible orientations of the loxF8 locus (wild-type and inverted orientation) is shown to the left (adapted from Lansing et al.^8^). Primers used for PCR to detect the orientation of the locus are displayed as green, blue, and red arrows. The position of the loxF8 sites and the distance between them are indicated. An agarose gel image of PCR products generated using the indicated primer combinations to detect the orientation of the loxF8 locus of the HEK293T^loxF8-zif^ cells is shown to the right. The HEK293T^loxF8-zif^ cells treated with GFP mRNA only were used as a wild-type control. A PCR reaction on DNA isolated from iPSCs derived from a patient carrying the int1h inversion was used as an inversion control. Primer combinations are indicated. M = Marker. The assays were performed three times (n = 3 biologically independent replicates). Source data

**Extended Data Fig. 9:**
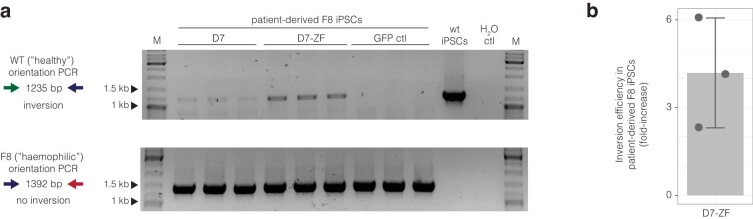
D7-ZF activity in patient-derived F8 hiPSCs. **a** Agarose gel image of PCR products generated using the indicated primer combinations to detect the orientation of the loxF8 locus in the F8 gene of the patient-derived F8 hiPSCs cells 48 h post transfection with D7 or D7-ZF mRNA. The cells treated with GFP mRNA were used as a wild-type control, the wild-type hiPSCs derived from a healthy donor were used as an inversion control. The experiment was done three times (n = 3, biologically independent samples, plotted as dots). Primer combinations are indicated. M = Marker. **b** Fold-increase of D7-ZF inversion efficiency in in patient-derived F8 hiPSCs over D7, quantified by qPCR. The experiment was done three times (n = 3, biologically independent samples, plotted as dots), the bar graph represents the mean value, the error bars indicate the standard deviation from the mean. Source data

To investigate the ability of D7-ZF to perform inversion on the genomic locus in the F8 gene, we transfected D7 and D7-ZF expression plasmids into HEK293T cells. HEK293T cells carry the F8 gene in the normal orientation, and successful recombination would invert it into the int1h disease orientation (Fig. 5e). Analysis of the genomic DNA (gDNA) by PCR-based assays revealed that the expression of the recombinases resulted in inversion of the loxF8 locus, with D7-ZF treatment improving the inversion efficiency noticeably (3.8-fold; Fig. 5f,g). Next, to evaluate the D7-ZF dependency on ZF DNA binding, we replaced the evolved ZFDs in D7-ZF with Zif268, thereby generating D7^278^–Zif268. We transfected D7-ZF and D7^278^–Zif268 mRNA into HEK293T reporter cells containing two loxF8 target sites flanked by Zif268 binding motifs (loxF8-5-zif(B)). Recombination of the loxF8-5-zif was detected only on the gDNA extracted from the samples transfected with D7^278^–Zif268 (Extended Data Fig. 8a), whereas inversion of the F8 locus was detected only on the gDNA extracted from the samples transfected with D7-ZF (Extended Data Fig. 8b). These results indicate ZF dependency of the D7-ZF heterodimer in human cells. Finally, D7-ZF mRNA transfection of patient-derived F8 int1h-hiPSCs led to a four-fold increase in inversion efficiency of the loxF8 locus over D7 (Extended Data Fig. 9a,b). The obtained results document the improved properties of D7-ZF, making it a preferred candidate for future therapeutic exploitation.

### ZFD–recombinase fusions with relaxed specificity

**Fig. 6:**
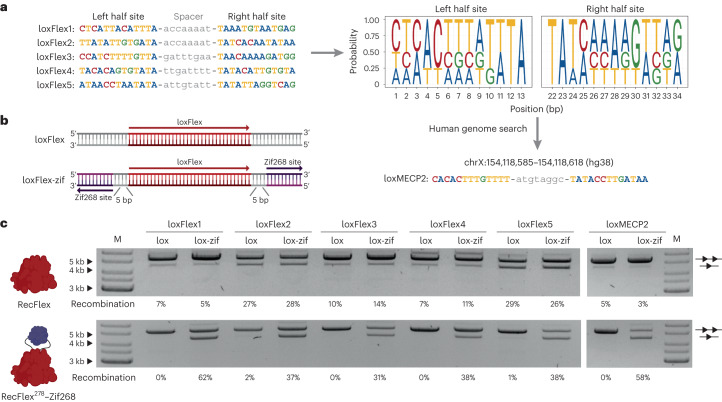
Target site specificity of RecFlex can be tailored by ZFD fusions. **a**, Alignment of target sites recombined by the RecFlex recombinase. Nucleotides of the half sites are highlighted in colors (adenine, blue; cytosine, red; guanine, green; thymine, yellow). Sequences of the spacers are shown in gray. A position probability matrix for the left and right half sites is shown to the right. A search using this matrix for potential therapeutic human genomic target sites revealed a candidate site on chromosome X (loxMECP2). **b**, Depiction of the target sites used for the activity assay. Activity of RecFlex and RecFlex^278^–Zif268 was tested on two types of target sites: loxFlex (34 bp) and the loxFlex-5-zif target sites where loxFlex is flanked by the Zif268 binding motif (9 bp) in orientation B (as shown on the depiction) relative to the lox site and spaced by the 5 bp. **c**, Representative plasmid-based activity assay of RecFlex and RecFlex^278^–Zif268. Activity was tested on the five loxFlex sites and on the human genomic loxMECP2 target site as well as on their lox-zif versions, in which the lox sites were flanked by the Zif268 binding motifs (spaced by the 5 bp, as shown in **b**). The upper band represents the unrecombined plasmid (line with two triangles), and the lower band represents the recombined plasmid (line with one triangle). The assay was performed three times (*n* = 3, biologically independent samples; replicates are shown in Source Data files). Recombination efficiencies were calculated from the ratio of the recombined and non-recombined band intensities, with mean recombination efficiencies indicated underneath the gel picture. M, marker. Source data

**Extended Data Fig. 10:**
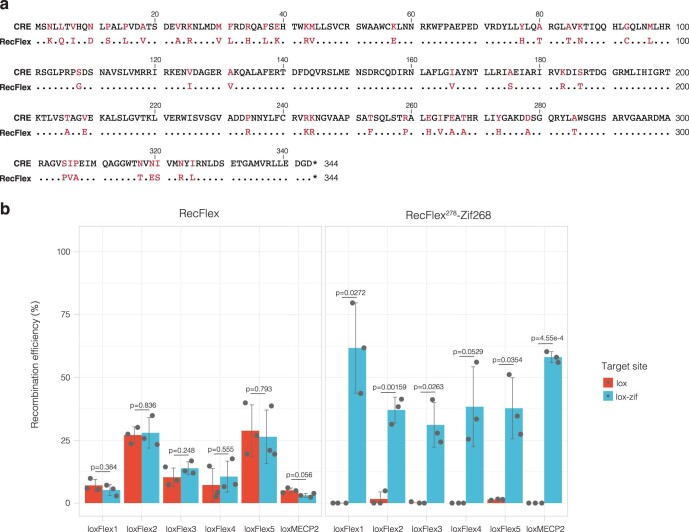
Amino acid sequence and recombination activity of RecFlex. **a** The amino acid sequence of RecFlex aligned to Cre (one-letter code) is shown. Dots indicate conserved residues; asterisks denote stop codons. **b** Quantification of the recombination rates for RecFlex and RecFlex-Zif268 fusion complex on five loxFlex sites, genomic loxMECP2 target site, and their lox-zif versions, in which the lox-sites were flanked by the Zif268 binding motifs. Recombination efficiencies were calculated from ratios of recombined and non-recombined band intensities shown in Fig. 6c. The assay was performed in triplicates (n = 3), plotted as dots. The bar graphs represent mean values, the error bars indicate the standard deviation from the mean. Statistical relevance of the triplicates was assessed using an unpaired two-sided t-test. P-values are indicated.

The findings presented offer important insights into the development of improved engineered recombinases, although their speed in generating custom enzymes that target new sequences remains suboptimal. In an effort to overcome this limitation, we explored the possibility of taming a relaxed specificity Cre-derived recombinase by introducing an insertional ZFD. To demonstrate this concept, we selected RecFlex, a recombinase identified from a previous evolution campaign, which displays relaxed target site specificity (Extended Data Fig. 10a). RecFlex is capable of recombining a range of lox-like sites, including loxFlex1, loxFlex2, loxFlex3, loxFlex4 and loxFlex5, which differ by 6 to 9 bp per half site (Fig. 6a). These target sites exhibit only 31–54% sequence similarity to each other, suggesting that RecFlex has the potential to recombine thousands of different target sequences. Through an extensive genome-wide investigation of loxFlex motif occurrences within the human genome, we successfully pinpointed a clinically relevant RecFlex-like target site situated within the MECP2 locus (loxMECP2) on the human X chromosome (Fig. 6a). Duplication events at this specific genomic locus were directly implicated in the onset of the MECP2 duplication syndrome, underscoring its therapeutic potential^33,34^.

To investigate whether the specificity of RecFlex can be redirected by insertional fusion with Zif268, we generated a RecFlex^278^–Zif268 fusion protein and assessed its activity on the five loxFlex sites with and without flanking zif-motifs (Fig. 6b). The RecFlex^278^–Zif268 fusion disrupted activity on all five lox sites, whereas recombination activity was restored in the presence of zif-motifs flanking these sites (ranging from 37% to 62%; Fig. 6c and Extended Data Fig. 10b). RecFlex^278^–Zif268 also disrupted the activity on the loxMECP2 target, which was restored only in the presence of zif-motifs flanking loxMECP2 (Fig. 6c and Extended Data Fig. 10b). Notably, RecFlex^278^–Zif268 activity was increased at all lox-zif target sites compared to wild-type RecFlex, suggesting not only that the enzyme was dependent on ZF binding to the flanking zif268 motif but also that the overall activity of the enzyme was enhanced, possibly by increased affinity for DNA provided by the ZFD. Our results suggest that this approach could enable the programming of engineered recombinases with partially tailored specificity within a shorter timeframe, by fusing a relaxed-specificity recombinase with an insertional ZFD.

## Discussion

Fully programable recombinases have long been sought after^13–19^ and have been described as the ultimate genome editing tool^35^. In the present study, we developed an approach to generate ZFD-dependent recombinases, offering a substantial advancement toward realizing this pivotal objective.

To build the fusion protein architecture, we screened over 1,700 variants of combinations of linker length, spacing and orientation of the lox-zif target sites. Optimal combinations of ZFD fusions on the N-termini or C-termini of recombinases did not impact activity of the enzymes on their lox sites but led to a prominent increase of recombination efficiency of up to 10-fold on the lox-zif sites. We assume that the enhanced activity of the ZF–recombinase fusions is due to the higher binding affinity of the resulting complex to DNA, which is achieved by extending the binding site from 34 bp to 52 bp (in the case of three-finger ZFDs). Despite the extended sequence recognition, the protein size of only approximately 500 amino acids makes this genome engineering tool highly economical. The application of these fusions could be advantageous for evolved recombinase variants with moderate activity, potentially leading to faster recombinase generation by reducing the number of evolution cycles required to achieve the desired activity level. However, as these fusion complexes remain active without the ZFD binding to its target, their off-target potential must be evaluated carefully.

Insertion of a ZFD into the coding sequence of a recombinase effectively impairs the recombinase’s activity on its lox sites until the ZFD binds to its cognate sites flanking the lox sites. Based on our results, we hypothesize that, in the absence of their binding motifs, the ZFDs interfere with the recombinase’s function or folding, leading to a disruption of the recombination activity. However, when the ZFDs are bound to the DNA outside the lox sites, the recombinases can form a functional tetramer and catalyze the recombination reaction with high specificity. As a result, our insertional fusion strategy enables superior target site specificity of the complex, ensuring that gene editing occurs only when both the ZFD and the recombinase are bound to their respective sites. We anticipate that the approach should be expandable to other DNA-binding domains^36^ and to other enzyme classes, such as transposases^37–40^, topoisomerases^41,42^ and integrases^43,44^.

We demonstrate that our approach is potentially versatile, as it can be applied to different recombinases and ZFDs. To illustrate its potential therapeutic value, we applied this approach to the heterodimeric D7 recombinase, which can be used to correct the large 140-kilobase (kb) int1h inversion responsible for severe hemophilia A (ref. ^8^). To target the genomic locus flanking the loxF8 target sites, we employed publicly available design tools to generate artificial ZFDs, which uncovered current limitations of these tools. The challenging nature of ZFD design has long been recognized, with substantial efforts from multiple research groups in this area^29,30,45–49^. These limitations have hindered the widespread application of ZFDs, as reliable prediction of active ZFDs remains a challenge. However, this is beginning to change with the release of the ZFDesign tool^50^ to aid in silico design of ZFDs.

Our findings have considerable implications for future genome editing applications, particularly in the development of engineered recombinases with relaxed specificity that can target a multitude of sites. Our relaxed specificity recombinase RecFlex is a prototype of such an enzyme. Although RecFlex currently falls short of being a fully reprogrammable recombinase, we envision that screening of libraries of relaxed-specificity recombinases will identify enzymes that can cover any sequences in the human genome and can be combined with inserted ZFDs to generate advanced genome editing enzymes.

## Methods

### Molecular cloning methods

All oligonucleotides were purchased from Sigma-Aldrich and are listed in Supplementary Table 4. Except for the ZF-SLiDE (described below), all PCR for cloning was performed with high-fidelity Herculase II Phusion DNA polymerase (Agilent, 600675); the cycling programs are listed in Supplementary Table 5. All restriction enzymes were purchased from New England Biolabs (NEB). An ISOLATE II PCR and Gel Kit (Bioline, Meridian Bioscience, BIO-52060) was used for purification of the PCR products and DNA fragments isolated from agarose gels. T4 DNA Ligase (Thermo Fisher Scientific, EL0011) was used for ligation reactions, and 2 µl of the ligation reaction was directly transformed into electrocompetent XL1-blue *E. coli* bacteria. In the case of libraries, the ligation reaction was membrane purified (MF-Millipore, GSWP01300), and 4 µl of the ligation was used for transformation. The transformed bacteria were grown overnight in LB medium with addition of antibiotic (30 µg ml^−1^ chloramphenicol for pEVO, 30 µg ml^−1^ chloramphenicol and 15 µg ml^−1^ kanamycin for pEVO containing Entranceposon during the pentapeptide scanning mutagenesis and 100 µg ml^−1^ ampicillin for pIRES, pEF1a and pCAGGs plasmids) and with addition of ʟ-arabinose, when induction of recombinase or ZF–recombinase from pEVO was of interest. Plasmids were purified from the overnight cultures using a GeneJET Plasmid Miniprep Kit (Thermo Fisher Scientific, K0503). Sequence verification was done with Sanger sequencing (Microsynth).

### Plasmids

The plasmid vectors used for the tests in bacteria were based on pEVO (described previously in refs. ^2,5,7^). The target sites were cloned as described previously^7^. In brief, primers containing the target sites of interest, BglII restriction site and an overlap with the pEVO plasmid (primers 1–106) were designed. These primers were used to produce a PCR product from a pEVO plasmid, which was subsequently cloned into a BglII-digested pEVO vector using a ColdFusion Cloning Kit (Systems Bioscience). For the target site libraries, each target site was cloned one by one, and the plasmids were mixed together in equal ratios. In pEVO, the recombinase or ZF–recombinase complex was cloned between BsrGI and XbaI or SacI and SbfI restriction sites. Dimer recombinases were cloned between SacI and XhoI (left monomer) and BsrGI and XbaI (right monomer). A Shine–Dalgarno sequence is located in front of each recombinase gene, which, in the case of the dimer, allowed bicistronic expression of both recombinases. In pEVO, expression of a recombinase or ZF–recombinase fusion complex is induced by arabinose promoter (araBAD). Different ʟ-arabinose (Sigma-Aldrich, A3256) concentrations were used for adjusting expression levels of the proteins (from 1 µg ml^−1^ to 200 µg ml^−1^).

Zif268 and ZFCCR5L genes were assembled by using a polymerase cycling assembly method (primers 107–120 and 121–133 were used), or, for some of the fusions, the sequence of Zif268 was produced by Twist Bioscience. The designed ZFDs were produced by Twist Bioscience.

For the N-terminal and C-terminal fusions, the linker libraries were created by cloning the sequences of different number (2, 4, 6, 8, 10 and 12) of GGS repeats between XhoI and BsrGI restriction sites in the pEVO plasmid. For this, we designed oligonucleotides containing the linker sequence and the sticky ends of the respective restriction sites (primers 144–155). These oligonucleotides were annealed at 95 °C for 10 min, followed by incubation at 23 °C for 20 min, to obtain the double-stranded DNA fragments and were directly used for ligation with the digested pEVO vector. The obtained plasmids with the linkers were mixed together in equal ratios to create a linker library. This library was used for a two-step cloning. In the case of the N-terminal fusion, first, Brec1 was cloned between the BsrGI and XbaI restriction sites, followed by the cloning of Zif268 sequence without stop codon between the SacI and XhoI restriction sites. For the C-terminal fusion, first, Zif268 was cloned between the BsrGI and XbaI restriction sites, followed by the cloning of Brec1 sequence without stop codon between the SacI and XhoI restriction sites. For the insertional fusion library, first, the XhoI and BsrGI restriction sites were introduced between the residues 278 and 279 of Brec1 by overlap extension PCR (primers 162 + 164 and 163 + 165). Then, the linker library was created by doing a PCR with a mix of primers containing different numbers (from 1 to 8) of GGS repeats, overlap with Zif268 and XhoI (for the forward primers) or BsrGI (for the reverse primers) restriction sites (primers 166–181). The digested PCR product of the Zif268 flanked by the linker libraries was subsequently cloned into pEVO_Brec1 vector between residues 278 and 279 via XhoI and BsrGI restriction sites. During the cloning of libraries, a coverage of at least 100,000 clones was reached.

For cloning of single ZF–recombinase fusion complexes, either the same cloning strategy as described for the libraries was employed or the modified pEVO vectors were used. For construction of the pEVO-N-Zif268-(GGS)12 vector, the DNA fragment flanked by BsrGI and XbaI restriction sites was produced by Twist Bioscience, in which the Zif268 is fused with the (GGS)_12_ linker, and this sequence is followed by BsiWI and XbaI restriction sites that can be used for the in-frame cloning of the recombinase. The Zif268 sequence is flanked by BsrGI and BbvCI restriction sites, which allows to exchange the ZFD in the fusion construct. For construction of the pEVO-(GGS)12-Zif268-C vector, in a similar manner, the DNA fragment flanked by BsrGI and XbaI restriction sites was produced by Twist Bioscience. In this case, BsrGI and SpeI (has compatible sticky ends with XbaI) restriction sites that allow cloning of the recombinase, were followed by the (GGS)_12_ linker sequence and the Zif268 gene, flanked by BbvCI and XbaI restriction sites. The produced fragments were cloned into the pEVO vector via BsrGI and XbaI restriction sites for construction of these plasmids. In the pEVO-N-Zif268-(GGS)12 vector, the recombinases were cloned between BsiWI (has compatible sticky ends with BsrGI) and XbaI restriction sites. For cloning into the pEVO-(GGS)12-Zif268-C vector, the recombinases were first amplified with a reverse primer that removes the stop codon from its coding sequence and cloned via BsrGI and SpeI restriction site. For the insertional fusion, by using overlap extension PCR, BbvCI and PspOMI restriction sites were introduced between residues 278 and 279 of the recombinase cloned into pEVO between BsrGI and XbaI restriction sites. The primers with the overhangs containing the BbvCI and PspOMI restriction sites and the (GGS)_8_ linker sequences were used for amplifying the Zif268 or designed ZFPs, and the digested PCR product was cloned into the recombinase sequence. The construct RecFlex^278^–Zif268 was produced by Twist Bioscience.

For transient expression of Brec1 or Brec1–Zif268 in HEK293T cells, these genes were cloned via BsrGI and XbaI restriction sites into the previously described pIRES-NLS-EGFP vector^7^ (Fig. 2g). For the transient expression of D7 or D7-ZF heterodimer in HEK293T cells, the monomers were cloned via BsrGI and XbaI restriction sites into a mammalian expression vector (pEF1a-mTagBFP-P2A-NLS-RecL or pEF1a-EGFP-P2A-NLS-RecR). In this vector, the recombinase or ZF–recombinase complex was translationally linked with mTagBFP or EGFP using a P2A self-cleaving peptide sequence, and the expression of this construct was driven by EF1a promoter.

The pCAGGs-lox-pA-lox-mCherry reporter plasmid was generated as previously described^7–9^ (Fig. 2g). In brief, the loxBTR, loxBTR-5-zif (A) and loxBTR-5-zif (B) target sites were introduced by PCR with overhang primers (primers 187–192) and were cloned into the pCAGGs vector via SalI and EcoRI restriction sites.

The pLentiR-loxF8-zif (SFFV-loxF8-zif-PURO) reporter lentivirus plasmid was generated from the pLentiCRISPR v.2 lentiviral backbone, a gift from F. Zhang (Harvard University) (Addgene plasmid 52961 (ref. ^51^); RRID: Addgene_52961)^52^, as described previously^8,9^. The loxF8-5-zif (B) target sites were introduced by PCR with overhang primers (primers 193 + 194) and were cloned via EcoRI and AgeI.

### Pentapeptide scanning mutagenesis and selection of the active mutated recombinase variants

Pentapeptide scanning mutagenesis was done using a Mutation Generation System Kit (Thermo Fisher Scientific), according to the manufacturer’s instructions. In brief, the M1-KanR Entranceposon was inserted into the pEVO containing the recombinase. To select for the variants where the transposon was inserted within the recombinase sequence, plasmid DNA of the obtained libraries was digested with BsrGI and XbaI restriction enzymes, and a DNA fragment that indicated successful integration of the transposon into the recombinase sequence (around 2 kb) was extracted and subcloned into a fresh pEVO vector. Next, the Entranceposon was removed from the library by NotI restriction digestion, and the mutated library containing five-amino-acid in-frame insertions throughout the recombinase sequence was cloned into the pEVO vectors containing recombinase-respective lox sites and induced by arabinose supplement to the medium. At this step, to confirm randomness of the mutations, single clones were sequenced and analyzed for recombination activity by PCR (described in the subsection ‘PCR for assessing recombination activity’). To select only the mutated recombinase variants that retained recombination activity, 500 ng of the induced library plasmid DNA was digested with NdeI and AvrII, which are located between the two lox sites on the pEVO plasmid. Thereby, the variants that did not excise the DNA sequence between the two lox sites were digested and removed from the pool, whereas the plasmids carrying the active variants remained intact. The digested library was then membrane purified, transformed and grown overnight with arabinose supplement. The next day, 500 ng of the active library DNA was again digested with NdeI and AvrII, and 25 ng of the digested DNA was used as a template for high-fidelity PCR (primers 156 + 157) to amplify the active mutated recombinases, which were digested with BsrGI and XbaI and subcloned to a fresh pEVO vector containing the respective lox sites and induced with arabinose. The selection cycle was repeated twice. At the final step, the plasmid DNA of active mutated recombinase libraries was extracted and prepared for long-read PacBio sequencing.

### Deep sequencing

Long-read PacBio sequencing of the active libraries of the mutated Cre, Brec1, D7L and D7R after the pentapeptide scanning mutagenesis was performed as previously described by Schmitt et al.^53^.

Nanopore sequencing of the active libraries of the mutated Vika, Vika2 and Vika3 recombinases after the pentapeptide scanning mutagenesis was performed in the following way. The plasmid DNA of the obtained libraries of the active mutants of Vika and Vika-like recombinases was extracted, and fragments containing the recombinases and target sites were obtained by digesting with BsrGI and ScaI restriction enzymes and subsequent gel extraction. Preparation of the libraries was performed following the protocol ‘Native barcoding amplicons’ using an SQK-LSK110 and an EXP-NBD104 kit (Oxford Nanopore Technologies). The three libraries were mixed before the preparation in a 1:1:1 ratio. Sequencing was performed on a MinION Mk1B nanopore sequencer with a FLO-MIN106 r9.4.1 flowcell (Oxford Nanopore Technologies).

The high-throughput screen for testing different combinations of linker and spacing lengths to develop the ZF–recombinase fusion architecture was performed as follows. The libraries of Brec1–Zif268 fusions were cloned to the pEVO target site library, transformed into XL1-blue *E. coli* and grown for 14–16 h in LB supplemented with chloramphenicol, and Brec1–Zif268 expression was induced by ʟ-arabinose (1 µg ml^−1^ for the N-terminal and C-terminal fusion and 200 µg ml^−1^ for the insertional fusion). The plasmid DNA was extracted, and fragments containing Brec1–Zif268 fusion complexes and target sites were obtained by digesting with SacI and ScaI restriction enzymes and subsequent gel extraction. Preparation of the libraries was performed following the protocol ‘Native barcoding amplicons’ using the SQK-LSK110 and the EXP-NBD104 kit (Oxford Nanopore Technologies). The three libraries (N-terminal fusion, C-terminal fusion and insertional fusion) were mixed before the preparation in a 1:1:5 ratio. Sequencing was performed on the MinION Mk1B nanopore sequencer with the FLO-MIN106 r9.4.1 flowcell (Oxford Nanopore Technologies).

### Deep sequencing analysis

PacBio HiFi DNA sequences after the pentapeptide scanning mutagenesis screen for Cre-type recombinases were aligned to the wild-type DNA reference sequence (Brec1, Cre, D7L or D7R) using Exonerate (version 2.3.0) with the ‘affine:bestfit’ model. From this alignment, the CIGAR values for each read were processed with a custom R script that counts 15-bp insertions for each position (R version 4.1.1 with tidyverse package version 1.3.1; ref. ^54^).

All nanopore sequencing data were basecalled with Guppy (Oxford Nanopore Technologies, version 5.0.7) in high accuracy mode. Only reads with a Phred quality score of 10 or greater were retained for further processing.

Sequencing reads from the pentapeptide scanning mutagenesis screen for Vika-type recombinases were aligned in two phases. In the first demultiplexing phase, all reads were aligned to sequences of backbones containing Vika, Vika2 and Vika3 and their respective target sites in unrecombined and recombined variants—six reference sequences in total. Subsets of reads unambiguously mapping to each of the references were individually subjected to the second alignment phase, in which each subset was mapped to a library of corresponding recombinase sequences containing pentapeptide insertions at each possible position. The final recombination rates of each protein variant were obtained by calculating a fraction of counts of reads mapped to a recombinase variant with recombined target sites to counts of all reads mapped the recombinase variant with either recombined or unrecombined target sites.

Sequencing reads from the high-throughput screen for testing different combinations of linker and spacing lengths were aligned to all possible sequence combinations (ZF fusion type, linker length, spacing between the loxBTR and zif268 target sites, recombined or non-recombined target sites) using minimap2 (version 2.17; ref. ^51^) with the ‘secondary’ option set to ‘no’. The alignment file was then filtered with SAMtools (version 1.11; ref. ^55^) using the view command and the -L option to only include reads that cover the target site and the recombinase by supplying BED files that contain specific coordinates for these regions. Relevant information from this alignment file was then extracted using GNU Awk (version 5.1.1) and processed and visualized in R (version 4.1.1 with tidyverse package version 1.3.1; ref. ^54^). The recombination rates were calculated by counting the number recombined and non-recombined reads for each ZF–recombinase complex and target site combination.

### PCR for assessing recombination activity

For a quick clonal analysis, the recombination activity was assessed by a PCR-based assay, described in Lansing et al.^7,8^. In brief, after the transformation, the recovery was plated on agar with chloramphenicol (15 µg ml^−1^). Single colonies were picked and grown in 500 µl of LB in the presence of chloramphenicol and ʟ-arabinose in a 96 deep-well plate for 16 h. Then, 1 µl of the grown cell suspension was used for colony three-primer PCR with MyTaq Polymerase (Bioline, BIO-21106) (primers 198–200) (Extended Data Fig. 5b). Primer 198 binds between two lox sites, and primer 199 binds upstream of the lox sites. Therefore, this primer combination generates a PCR product of ∼500 bp, indicating the non-recombined substrate. Primer 200 binds downstream of the second lox site, and a combination with primer 199 will generate a shorter ∼400-bp product, indicating the recombined plasmid. Short elongation time used for this PCR reaction does not allow product generation from the non-recombined template (∼1,140 bp) for this primer combination.

### Plasmid-based recombination test in bacteria

To asses recombination activity, the efficiency of the excision of the two target sites on the pEVO plasmid was evaluated. For this, the expression of the recombinase or ZF–recombinase complex in the overnight cultures was induced by addition of ʟ-arabinose. Testing of the fused ZF–recombinase complexes and recombinases alone on the same target sites was always performed at the same concentration of ʟ-arabinose, but the induction levels varied between the different experiments, as described here. Testing of the N-terminal and C-terminal fusions for Brec1–Zif268 and Brec1–ZFCCR5L were performed at 1 µg ml^−1^ and, for RecHTLV–Zif268, at 10 µg ml^−1^. This low induction level was used to demonstrate the enhanced recombination efficiencies when recombinases were fused to the ZFD on the lox-zif target sites. The insertional fusions were tested at different concentrations, depending on the activity of the non-fused recombinase on the respective lox sites (200 µg ml^−1^ was used for Brec1-loxBTR, D7R-loxF8R and RecFlex-loxMECP2; 100 µg ml^−1^ was used for RecHTLV-loxHTLV, D7L-loxF8L, D7-loxF8 and all the off-targets, RecFlex-loxFlex1, RecFlex-loxFlex4, Vika2-vox2, Vika3-vox3 and Vika4-vox4; and 10 µg ml^−1^ was used for RecFlex-loxFlex2, RecFlex-loxFlex3 and RecFlex-loxFlex5). Next, 500 ng of the plasmid DNA extracted from the induced cultures was digested with BsrGI and XbaI or SacI and SbfI restriction enzymes. Then, 200 ng of the digested DNA and 5 µl (2.5 µg) of GeneRuler DNA Ladder Mix (Thermo Fisher Scientific, SM0331) as a loading control were loaded to a 0.8% agarose gel stained with RedSafe (Intron Biotechnology, 21141), and the gel was run at 70 V for 90 min. Three bands could be seen on the agarose gel after the gel electrophoresis. The smallest band of 1 kb shows the recombinase (∼1.5 kb for the recombinase fused with a ZFD, ∼2 kb for the recombinase heterodimer (D7) and ∼3 kb for the recombinase heterodimer fused with ZFP (D7-ZF)) and is used as a control for the presence of the tested recombinase or complex in the digested plasmid pool. The biggest band of ∼5 kb shows the unrecombined pEVO backbone, and a smaller band of ∼4.3 kb shows the recombined substrate. The gel images were taken with an Infinity VX2-3026 transilluminator and InfinityCapt software (Vilber). The band intensities of the bands (5 kb and 4.3 kb) were calculated using Fiji (version 2.0.0.-rc-65/1.52a). The recombination efficiency was quantified by the ratio of the non-recombined and the recombined band intensities.

### ZFD design for genomic F8 locus

ZFDs were designed for the sequences upstream and downstream of the loxF8 target site in the human genome. Two three-finger ZFDs (ZFL1 and ZFL2) targeting the DNA sequence 5′-GCAATGAAT-3′ 5 bp upstream of the loxF8 target site (reverse strand) were designed using the publicly available platform of Persikov et al.^30^. Two three-finger ZFDs (ZFR1 and ZFR2) targeting the DNA sequence 5′-AAGATTGGC-3′ 5 bp downstream of the loxF8 target site (forward strand) and one three-finger ZFD (ZFR3) targeting the DNA sequence 5′-CAAGATTGG-3′ 4 bp downstream of the loxF8 target site (forward strand) were designed using the same publicly available platform^30^. One four-finger ZFD (ZFR4) targeting the DNA sequence 5′-CAAGATTGGCAG-3′ 4 bp downstream of the loxF8 target site (forward strand) was designed by modular assembly using the list of published ZF modules^29^. The amino acid sequences of the designed ZFDs are listed in Supplementary Table 1.

### ZF-SLiDE

Zinc-finger domains designed for the loxF8 flanking sequences were evolved based on the established substrate-linked directed evolution of recombinases^2,4,6–8^. SLiDE links excision activity of the lox sites by a recombinase to the plasmid that encodes its gene. Because the activity of the recombinase was induced by ZFD binding to its target sites next to the lox sites, we used this property for evolving the ZFDs in this system. A schematic of the procedure is depicted in Fig. 4a. We started by creating a library of the ZFDs by performing 50 cycles of error-prone PCR with primers 156 and 157 and MyTaq Polymerase (Bioline, BIO-21106), which lacks a proof-reading activity and, therefore, introduces mutations. The PCR product was digested with BbvCI and PspOMI, and the band of around 400 bp for the ZFL and around 500 bp for the ZFR (both cases included the ZFs and the flanking linkers) was extracted from an agarose gel. This insert containing a ZF library was cloned into the digested pEVO vectors that contained the loxF8L-flank or loxF8R-flank target sites and D7L or D7R recombinase sequences, respectively, with the frameshift insertion between amino acids 278 and 279 that is flanked by BbvCI and PspOMI restriction sites. XL1-blue *E. coli* was transformed with the pEVO libraries and grown in 100 ml of LB medium with chloramphenicol (30 µg ml^−1^) and ʟ-arabinose (200 µg ml^−1^, 10 µg ml^−1^ or 1 µg ml^−1^). Then, 10 ml of the culture was used for the plasmid extraction, and 500 ng of plasmid DNA was digested with NdeI and AvrII that are located between the two lox sites on the plasmid. Thereby, the inactive variants, which did not perform excision, were eliminated from the pool. The remaining active variants were amplified using error-prone PCR with primers binding upstream of the Rec-ZF gene and downstream of the target site (primers 195 + 196). The PCR product was digested with BbvCI and PspOMI to extract only the ZFD and the flanking linker sequences and was cloned in the pEVO into the intact, wild-type recombinase gene, as described above, thereby starting a new cycle of ZF evolution. Additionally, to prevent the evolving ZFD from gaining a generally relaxed specificity, we performed counter-selection on the loxF8L and loxF8R target sites, which did not have flanking ZF target sequences. For the counter-selection, the digested ZF library fragments were cloned into pEVO containing the D7L or D7R recombinase sequence and the loxF8L or loxF8R sites, respectively. In this case, a high ʟ-arabinose concentration was used (200 µg ml^−1^). After plasmid DNA extraction, we directly proceeded to error-prone PCR and amplified the inactive variants with the primers binding upstream of the ZF-recombinase gene and between the lox sites (primers 197 + 198). The cycling process was repeated with lowering ZF–recombinase expression levels (by lowering the concentration of ʟ-arabinose) on the flanked target sites to select for most improved variants and keeping it always high on the lox sites for the counter-selection. Overall, 17 cycles of evolution on the flanked target sites and three cycles of counter-selection evolution on the lox sites were performed for both ZFL and ZFR libraries. Finally, both recombinases fused with ZF libraries were combined, and the dimers inactive on the loxF8 target site (eight cycles) and active on the loxF8-flank sites (three cycles) were selected in a similar way, as described in Hoersten et al.^12^ and Lansing et al.^8^. High-fidelity Herculase II Phusion DNA polymerase (Agilent) was used for dimer selection to select the compatible combinations without introducing new mutations in the recombinase sequences.

### Sequence analysis of the evolved ZFDs

Active clones from the final ZFL (75 clones) and ZFR (59 clones) libraries were picked and sent for *E. coli* overnight Sanger sequencing (Microsynth). The obtained sequences were analyzed to determine the mutational changes in the ZFD and linker sequence, by comparing to the respective ZFL1 and ZFR4 sequences. The analysis of the sequencing data was performed in R version 4.1.0 using the dplyr, Sequence tools (https://github.com/ltschmitt/SequenceTools) and ggplot2 packages.

### Cell culture

HEK293T (American Type Culture Collection) cells were cultured in DMEM (Gibco, 10564011) with 10% FBS (Gibco, A5256701) and 1% penicillin–streptomycin (10,000 U ml^−1^, Thermo Fisher Scientific, 15140122) at 37 °C and 5% CO_2_ in a HERAcell Incubator 240i (Thermo Fisher Scientific). Trypsin-EDTA (Gibco, 25200056) was used for dissociation of the cells for splitting.

Patient-derived F8 hiPSCs were reprogrammed at the Stem Cell Engineering Facility of the Center for Molecular and Cellular Bioengineering (CMCB) at TU Dresden (described in Lansing et al.^8^). hiPSCs were cultured in StemFit Basic04 Complete Type (AJINOMOTO). The first 24 h after splitting, the medium was supplemented with 10 µM ROCK inhibitor (Y-27632, Tocris, 1254). Accutase (Thermo Fisher Scientific, 00-4555-56) was used for detachment of the cells for splitting. The coating was performed with iMatrix-511 silk laminin (NIPPI) according to the manufacturer’s instructions.

### Generation of the HEK293T^loxF8-zif^ cell line

HEK293T cells were transfected with the pLentiR-loxF8-zif-PURO plasmid, lentiviral gag/pol packaging plasmid (psPAX2, Addgene no. 12260) and the envelope plasmid VSV-G (pMD2.G, Addgene no. 12259), using standard polyethylenimine transfection. Forty-eight hours after transfection, viral particles generated in the supernatant were harvested and used to infect fresh HEK293T cells. Seventy-two hours after transduction, cells were exposed to selection with 2 µg ml^−1^ puromycin for 7 d. gDNA of the surviving cells was isolated and subjected to a reporter-specific PCR. Sequencing of the amplified fragment confirmed integration of the reporter construct in the genome.

### Cell culture plasmid recombination assay

To test activity of Brec–Zif268 fusion complexes, a plasmid assay in HEK293T cells was performed. In total, 30,000 HEK293T cells per well were seeded in a 96-well plate. The next day, 25 ng of the pIRES expression plasmid and 25 ng of the pCAGGs reporter plasmid were transfected using Lipofectamine 2000 Transfection Reagent (Thermo Fisher Scientific, 11668019). The cells were analyzed with a MACSQuant VYB (Miltenyi Biotec) 48 h after transfection. HEK293T cells were gated for single cells, for transfected population (GFP^+^ cells) and, finally, for the transfected cells that successfully performed the recombination of the reporter (mCherry^+^GFP^+^ cells). The recombination efficiency was calculated by the percentage of double-positive cells (mCherry^+^GFP^+^) divided by the percentage of all GFP^+^ cells.

To test the inversion efficiency of the genomic loxF8 locus, HEK293T cells were transfected with pEF1a expression plasmids expressing D7 or D7-ZF. For this, 200,000 HEK293T cells per well were seeded in a 12-well plate. The next day, 400 ng of pEF1a plasmid expressing D7L or D7L-ZFL and 400 ng of pEF1a plasmid expressing D7R or D7R-ZFR were transfected using Lipofectamine 2000 Transfection Reagent (Thermo Fisher Scientific, 11668019). Seventy-two hours after transfection, the cells were analyzed with the MACSQuant VYB and harvested. To determine transfection efficiency, HEK293T cells were gated for single cells and for transfected population (GFP^+^BFP^+^ cells). In both experiments, analysis of the flow cytometry data was performed using FlowJo 10 software (BD Biosciences).

### In vitro transcription

The DNA templates for in vitro transcription (IVT) were generated by PCR from the pEF1a plasmids with EGFP (primers 201 + 202), D7L, D7L-ZFL or D7L-Zif268 (primers 203 + 204), D7R, D7R-ZFR or D7R-Zif268 (primers 205 + 206). D7L, D7R, D7L-ZFL(G10), D7R-ZFR(G10), D7L-Zif268, D7R-Zif268 and eGFP mRNA were produced using a HiScribe T7 ARCA mRNA Kit (NEB, E2065S) and purified using a Monarch RNA Cleanup Kit (NEB, T2040L), according to the manufacturer’s instructions.

### mRNA transfection

HEK293T^loxF8-zif^ reporter cells and patient-derived F8 hiPSCs were transfected with IVT-produced mRNA using Lipofectamine MessengerMAX Transfection Reagent (Thermo Fisher Scientific, LMRNA015). HEK293T^loxF8-zif^ cells were seeded at a density of 300,000 cells per well in a 12-well format the day before transfection. Then, 300 fmol of mRNA per well (140 ng of D7L-Zif268 and D7R-Zif268 or 140 ng of D7L-ZFL and 150 ng of D7R-ZFR or 70 ng of eGFP mRNA) was used for transfection. F8 hiPSCs were seeded at a density of 600,000 cells per well in a six-well format the day before transfection. For each well, 740 fmol of recombinase mRNA (250 ng of D7L and D7R mRNA or 360 ng of D7L-ZFL mRNA and 380 ng of D7R-ZFR mRNA) and 50 ng of eGFP mRNA were used for transfection. In both cases, cells were analyzed 48 h after transfection by fluorescence microscopy and harvested.

### Detection of recombination by PCR on gDNA

gDNA from HEK293T cells and F8 hiPSCs transfected with D7 or D7-ZF was isolated using a QIAamp DNA Blood Mini Kit (Qiagen, 51106). The inversion of the 140-kb DNA fragment between the two loxF8 target sites was detected by PCR, as described previously by Lansing et al.^8^. Primer pairs 207 + 208 and 209 + 210 were used to amplify the WT (‘healthy’) orientation of the 140-kb fragment that can be detected in HEK293T cells or, in case of the inversion event, in F8 hiPSCs. Primer pairs 207 + 210 and 208 + 209 were used to amplify the inverted (‘hemophilic’) orientation of the int1h that is detected in F8 hiPSCs or, in case of the inversion event, in HEK293T cells. Recombination of loxF8-zif genomic reporter was detected by PCR using the primer pair 211 + 212 that amplifies both recombined (644 bp) and unrecombined (1,308 bp) fragments.

### Inversion quantification

Inversion efficiency was quantified using a qPCR-based assay as described previously by Lansing et al.^8^. In brief, to detect the WT orientation (inversion event in F8 hiPSCs), the primers 209 + 210 were used; to detect the inverted ‘hemophilic’ orientation (inversion event in HEK293T cells), the primers 206 + 207 were used. In both cases, a TaqMan amplicon specific probe was used. Samples of 1%, 5%, 10%, 25%, 50% and 100% inversion were generated by mixing gDNA of WT iPSCs and F8 hiPSCs at appropriate ratios. The Cq values of these mixtures were used to build a standard curve and extrapolate the inversion efficiency of the gDNA samples of interest. The calculated inversion efficiencies from the transfected HEK293T cells were normalized by transfection efficiencies. Because gDNA of male iPSCs (one X chromosome) was used for generation of the standard curve used in the quantification, the calculated inversion efficiencies from the transfected HEK293T cells (female, two X chromosomes) were divided by 2. For quantification in F8 hiPSCs, an average of the triplicate samples transfected with D7 was calculated, and the fold change of each replicate treated with D7-ZF was quantified using the following formula: (D7-ZF inversion − D7 inversion _average_) / D7 inversion _average_.

### ChIP-seq and qPCR validation

D7L-ZFL and D7R-ZFR were fused with EGFP and cloned in a modified version of the tetracycline-inducible plentiX vector (described previously in refs. ^8,9^). These plasmids were used as a template for PCR with the primers 201 + 204, 201 + 206 and 201 + 202. The obtained DNA templates were used for IVT, as described above. Two 10-cm dishes were seeded with four Mio HEK293T cells each. The next day, 6.5 µg of D7L-ZFL-EGFP and 6.5 µg of D7R-ZFR-EGFP or 3 µg of EGFP mRNA were transfected as described above. ChIP was performed as described previously^8,9^. In short, 24 h after transfection, cells were crosslinked with 1% formaldehyde for 10 min, and chromatin extraction and shearing were performed using a truChIP Chromatin Shearing Kit (Covaris) following the manufacturer’s protocol (high cell number), followed by chromatin shearing with a Covaris M220 sonicator. Then, 1% of the sheared chromatin was separated for further qPCR validation as an input sample, and the rest was used for immunoprecipitation. Sonicated chromatin was immunoprecipitated with a goat GFP antibody (MPI-CBG antibody facility, 1:5,000) and Protein G sepharose beads (Protein G Sepharose 4 Fast Flow, GE Healthcare). Eluates were reverse crosslinked, followed by RNA and protein digestion.

ChIP DNA sequencing was performed at the Novogene facility. The DNA fragments were repaired, A-tailed and further ligated with Illumina adapter. The final DNA library was obtained by size selection and PCR amplification. The library was checked with Qubit and real-time PCR for quantification and bioanalyzer for size distribution detection. Quantified libraries were sequenced on the Illumina platform, aiming for at least 30 million pairs of sequencing reads per sample, with each read being 150 bp long. Additionally, the same set of DNA samples was sequenced at the Deep Sequencing Facility of TU Dresden, using the same sample processing pipeline but a read length of 100 bp.

Reads obtained from both sequencing facilities were pooled and aligned to the human reference genome assembly GRCh38.p13 (ref. ^56^) using bwa-mem2 aligner^57^ and SAMtools^58^. Reads identified as PCR and optical duplicates were removed using the Picard MarkDuplicates tool, and the final peak calling was performed with Genrich^59^, using the ENCODE blacklist (version 2)^60^ for filtering out problematic regions. Visualizations of the ChIP-seq pile-up signals were generated with the USCS Genome Browser^61^, directly from the read alignment files after the duplicate removal step. All steps involving manipulations and comparisons of genomic intervals were done using BEDTools^62^.

De novo motif discovery was performed with MEME-ChIP script from the MEME suite^63^, which was executed with the following set of arguments: ‘-ccut 0 -seed 0 -meme-mod oops -minw 8 -maxw 30 -meme-nmotifs 10 -meme-minsites 20 -centrimo-local’. For the motif comparison stage, a database of loxP and loxF8 sequences was used, with addition of predicted zinc-finger DNA-binding motifs. To generate an input file, BEDTools^62^ were used to extract 500-bp-long sequences centered at the peak summits reported by the peak-calling pipeline.

Twenty-five high-confidence peaks were found for D7-ZF. As a comparison, 84 off-target sites were detected for the recombinases alone using the same cutoff^8^, indicating that fusion with the ZFDs did not increase the number of binding sites in the genome. Ten out of 25 peaks that were identified by ChIP-seq were additionally tested by qPCR for recombinase binding (primers 213–236). qPCR was performed using SYBR Green Master Mix (Thermo Fisher Scientific, ABsolute qPCR SYBR Green Mix, AB1159A), and the ChIP samples and input samples were compared.

Ten peak sequences were tested in a plasmid-based assay for recombination in bacteria. A sequence of 70 bp around each peak was chosen based on the position of the identified zinc-finger DNA-binding motifs. DNA insert of 70 bp for each peak was generated by PCR (primers 237–256) and cloned into the pEVO vector twice as target sites for excision, as described above. D7-ZF was expressed at 100 µg ml^−1^ ʟ-arabinose on the cloned sequences, and plasmid-based recombination tests in *E. coli* were performed, as described above.

### Recombinase and CRISPR–Cas9 deletion detection

To investigate potential unintended deletion of the 140-kb fragment on the F8 locus of D7 and D7-ZF recombinases, we tested them in HEK293T cells and compared recombinase approach with CRISPR–Cas9. Then, 600 fmol of mRNA per well (200 ng of D7L and D7R, 280 ng of D7L-ZFL and D7R-ZFR, 800 ng of Cas9 (TriLink BioTechnologies, L-7206) or 140 ng of eGFP mRNA) was used for transfection in a 12-well format. Cas9 mRNA was transfected in a combination with 8 pmol gRNA specific for the inverted repeat (5′-GGUCCCCGGGGUUGUGCCCC-3′), as published by Park et al.^32^. Genomic DNA was isolated 48 hours after transfection and analyzed for inversion and deletion events by PCR. Primer pair 207 + 208 was used to amplify the WT (‘healthy’) orientation of the 140-kb fragment, and primer pair 208 + 209 was used to amplify the inversion event in HEK293T cells. Primer pair 207 + 209 was used to amplify the potential deletion of the 140-kb fragment. The PCR product obtained from Cas9 and gRNA transfected samples was cloned into pMiniT 2.0 plasmid using an NEB PCR Cloning Kit, according to the manufacturer’s recommendations. After transformation into NEB 10-beta *E. coli*, nine colonies were picked and sequenced using *E. coli* overnight Sanger sequencing (Microsynth) with the ‘cloning analysis forward primer’, provided in the NEB PCR Cloning Kit.

### Off-target analysis

The position weight matricies (PWMs) for the evolved ZFL and ZFR in the selected D7-ZF clone were obtained using the Interactive PWM Predictor^64^. Potential genomic off-targets of D7 recombinases^8^ were then scanned for occurrences of the PWM motifs upstream or downstream of the lox sites using the FIMO tool from the MEME Suite^63^, using a *P* value threshold of 0.001. Reported results were filtered to ensure that coordinates of matches are within an expected distance of 4 bp to 6 bp from the corresponding ‘left’ or ‘right’ half site. The predicted off-targets are listed in Supplementary Table 1.

### Target site identification

To find a locus in the human genome that can be targeted by RecFlex, a human genome-wide search for loxFlex motif occurrences was performed using FIMO^63^.

### Reporting summary

Further information on research design is available in the Nature Portfolio Reporting Summary linked to this article.

## Online content

Any methods, additional references, Nature Portfolio reporting summaries, source data, extended data, supplementary information, acknowledgements, peer review information; details of author contributions and competing interests; and statements of data and code availability are available at 10.1038/s41587-023-02121-y.

## Supplementary information

Supplementary Information Supplementary Note 1 and Figs. 1–6.

Reporting Summary

Supplementary Tables 1–5.

## Source data

Source Data Figs. 1–6 and Extended Data Figs. 6, 8 and 9 Unprocessed gels of Figs. 1f,g, 2e,f, 3b, 4b, 5b–d,f and 6d and Extended Data Figs. 6e, 8 and 9a.

## Data Availability

The sequence data generated in this study are deposited in the Sequence Read Archive with accession number PRJNA1047027 (ref. ^65^). Source data are provided with this paper.

## Code availability

Custom R scripts, SequenceTools version 0.0.1 (https://github.com/ltschmitt/SequenceTools) and ZF-rec analyses (https://github.com/ltschmitt/ZF-rec_analyses) (ref. ^66^) were used in this study.
