# Supplementary material for: Activation of recombinases at specific DNA loci by zinc-finger domain insertions
Source: Nat Biotechnol. 2024 Jan 31;42(12):1844–54. doi: 10.1038/s41587-023-02121-y (PMC11631766; doi:10.1038/s41587-023-02121-y)
Supplement: Supplementary file 1 — Supplementary Note 1 and Figs. 1–6. [file 41587_2023_2121_MOESM1_ESM.pdf]

nature biotechnology

Article

https://doi.org/10.1038/s41587-023-02121-y

Activation of recombinases at specific DNA loci by zinc-finger domain insertions

In the format provided by the authors and unedited

Supplementary Information

Supplementary Note 1

Analysis of the 3D protein structure

To find a universal position in the recombinase sequence for insertional fusions in Cre-type recombinases, we considered the most frequent positions that were found to be tolerated in the pentapeptide mutagenesis screen in all four recombinases: aa14, aa64, aa278, aa323, and aa328. We inspected the crystal structure of the Cre/loxP synapse pre-cleavage complex (PDB ID 1Q3U, ref.[1]) to nominate the best position for the ZF insertion. We used the 3D Protein imager for the analysis[2]. All currently available experimental structures of Cre are lacking information about its N-terminal tail (aa 1-20), therefore the position aa14 was excluded from the analysis. Visual inspection of the selected positions revealed that residues N323 and L328 are positioned in an area of extensive protein-protein interaction of the C-terminal domains of the monomers, while residues F64 and D278 are located on the exposed surface of the dimer complex and are not involved in protein-protein or protein-DNA interactions (Supplementary Fig. 2a). Next, we estimated the distance from these residues to the nucleotide following the loxP target site, which amounted to 52.2 Angstroms for F64 and 30.4 Angstroms for D278 (Supplementary Fig. 2b). According to the spatial accessibility and closer proximity to the DNA, the position in the recombinase sequence between residues 278 and 279 was selected for the insertional fusion of a zinc finger protein.

To find a universal position in the recombinase sequence for insertional fusions in Vika-type recombinases we first generated a 3D model of wt Vika using the online tool ColabFold, which predicts protein structure using AlphaFold2 and Alphafold2-multimer, and protein alignment and templates using MMseqs2 and HHsearch.

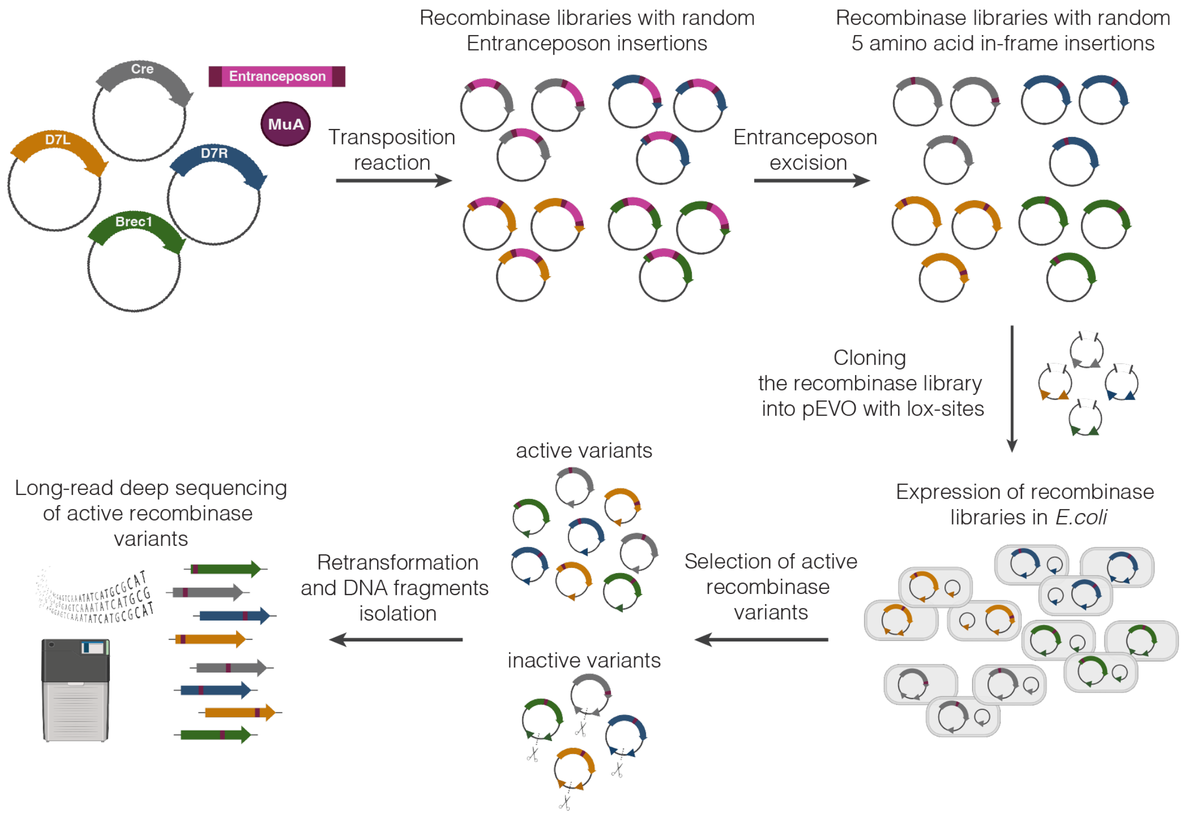

Supplementary Figure 1

Pentapeptide scanning mutagenesis screen.

Schematic representation of the pentapeptide scanning mutagenesis procedure. The recombinases (Cre, D7L, D7R, Brec1) cloned into the pEVO vector were combined with the MuA transposase and Entranceposon for the transposition reaction that resulted in the library, in which the Entranceposon was randomly inserted within the recombinase sequence. In the next step, the inserted Entranceposon was excised by a restriction enzyme digest, resulting in the library of recombinase mutants12/18/23 11:24:00 AM carrying five amino acid in-frame insertions. The obtained library was cloned into pEVO plasmids carrying the respective recombinase target sites for selection. Upon expression of the recombinases, the mutated variants retaining recombination activity on the target sites were selected by digestion with a unique restriction enzyme (depicted as scissors) and sequenced with PacBio long-read sequencing.

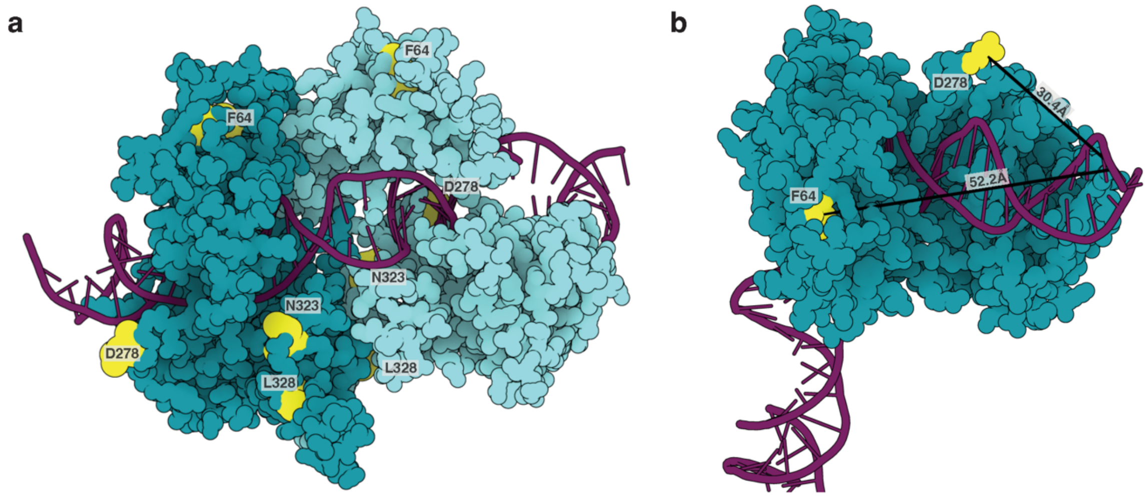

Supplementary Figure 2

3D model of positions allowing pentapeptide insertions.

a A dimer of the Cre/loxP synapse pre-cleavage complex (PDB ID 1Q3U, ref.[1]) is shown. The DNA is shown in dark magenta, and the two Cre monomers are highlighted by dark and light turquoise. The most frequent positions that tolerated insertions in the pentapeptide scanning mutagenesis of the Cre-type recombinases are highlighted in yellow with positions numbered. **b** A monomer of the Cre/loxP synapse pre-cleavage complex (PDB ID 1Q3U, ref.[1]) is shown. The distances between selected residues and the DNA (to the nucleotide following the loxP target site) are indicated. Both images (**a**) and (**b**) were created using the 3D Protein imager(ref.[2]).

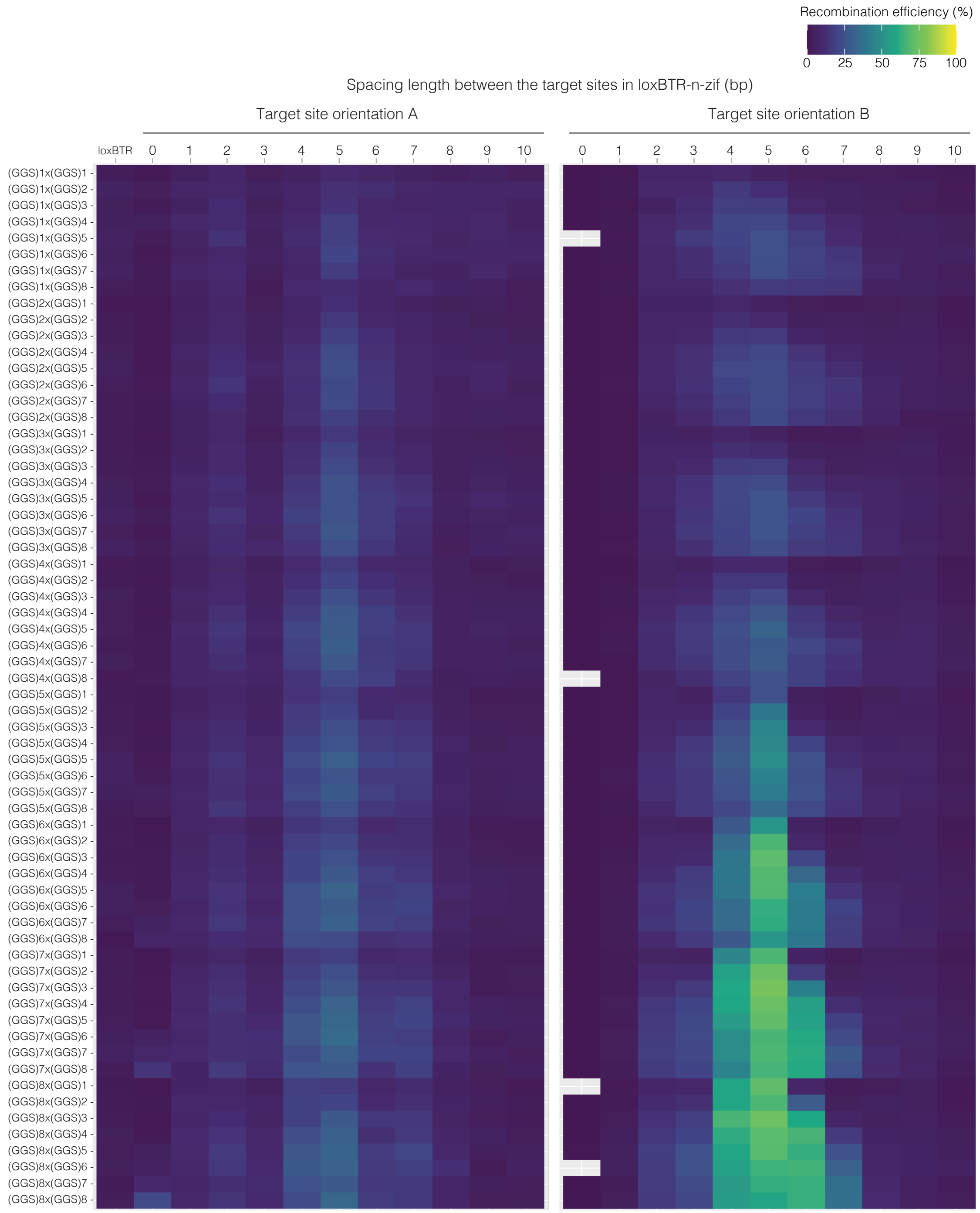

Supplementary Figure 3

Results of the deep-sequencing recombination screen for insertional Brec1-Zif268 fusions. Heatmap deduced from the nanopore-sequencing results for the insertional Brec1-Zif268 fusions. The Gly-Gly-Ser linker length is shown on the y-axis (left linker **x** right linker).

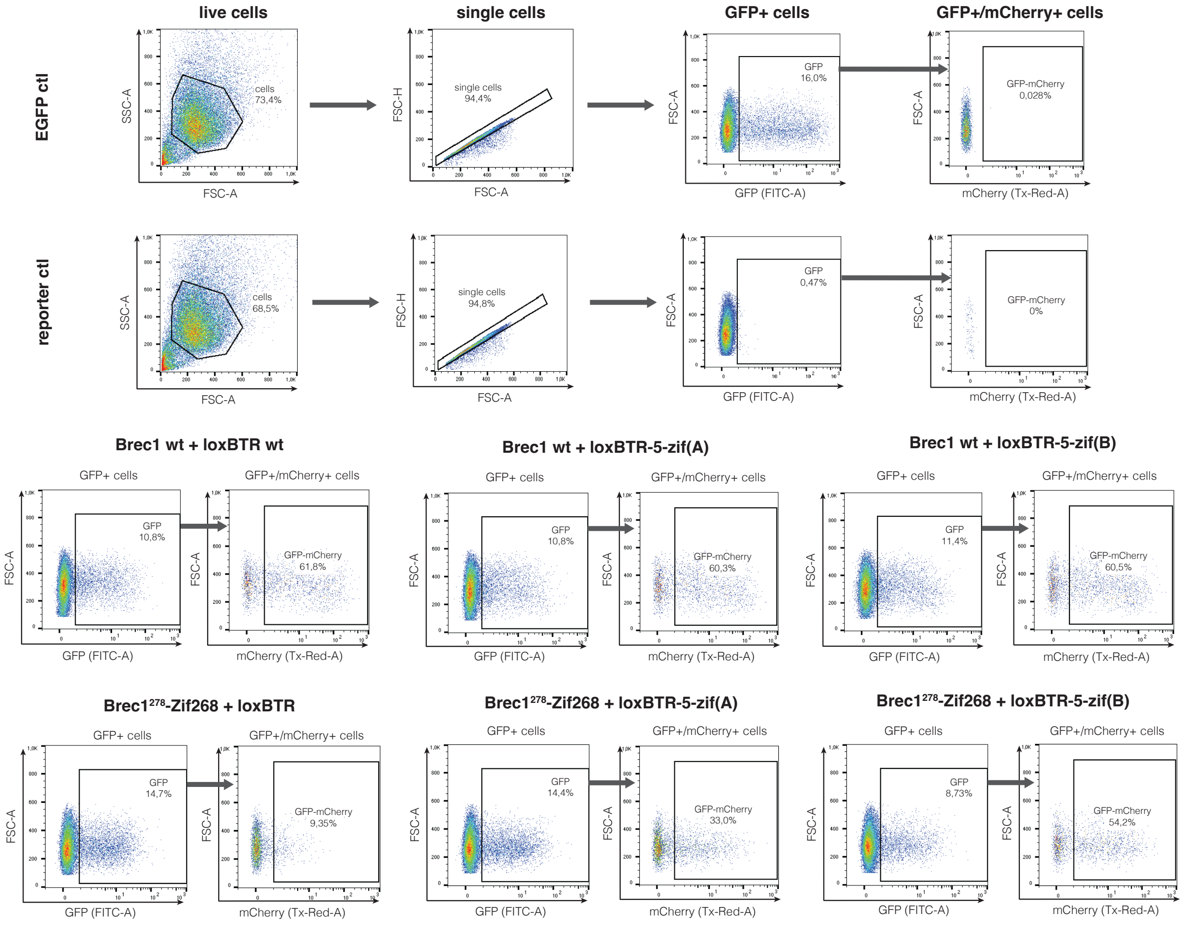

Supplementary Figure 4

Activity of Brec1 and Brec1^{278}-Zif268 in transient reporter assay in human cells.

Representative dot plots obtained by flow cytometry of HEK293T cells transfected with the loxBTR, loxBTR-5-zif (A) or loxBTR-5-zif (B) reporter constructs and the Brec1 or Brec1^{278}-Zif268 expression constructs. The expression construct encodes the EGFP gene fused with the recombinase or ZF-recombinase. The reporter constructs express mCherry upon successful recombination of the target sites. On the top, the gating strategy is shown for the samples, in which the empty expression construct only (EGFP ctl) or loxBTR wt reporter only (reporter ctl) were transfected. HEK293T cells were gated for live cells, for single cells, for transfected population (GFP+ cells, the GFP fluorescent signal was measured using a blue 488 nm laser and a 525/50 nm emission filter), and finally for the transfected cells that successfully performed recombination of the reporter (mCherry+GFP+ cells, the mCherry fluorescent signal was measured using a yellow 561 nm laser and a 615/20 nm emission filter). For the other samples only the dot plots for the GFP+ and mCherry+GFP+ gating is shown.

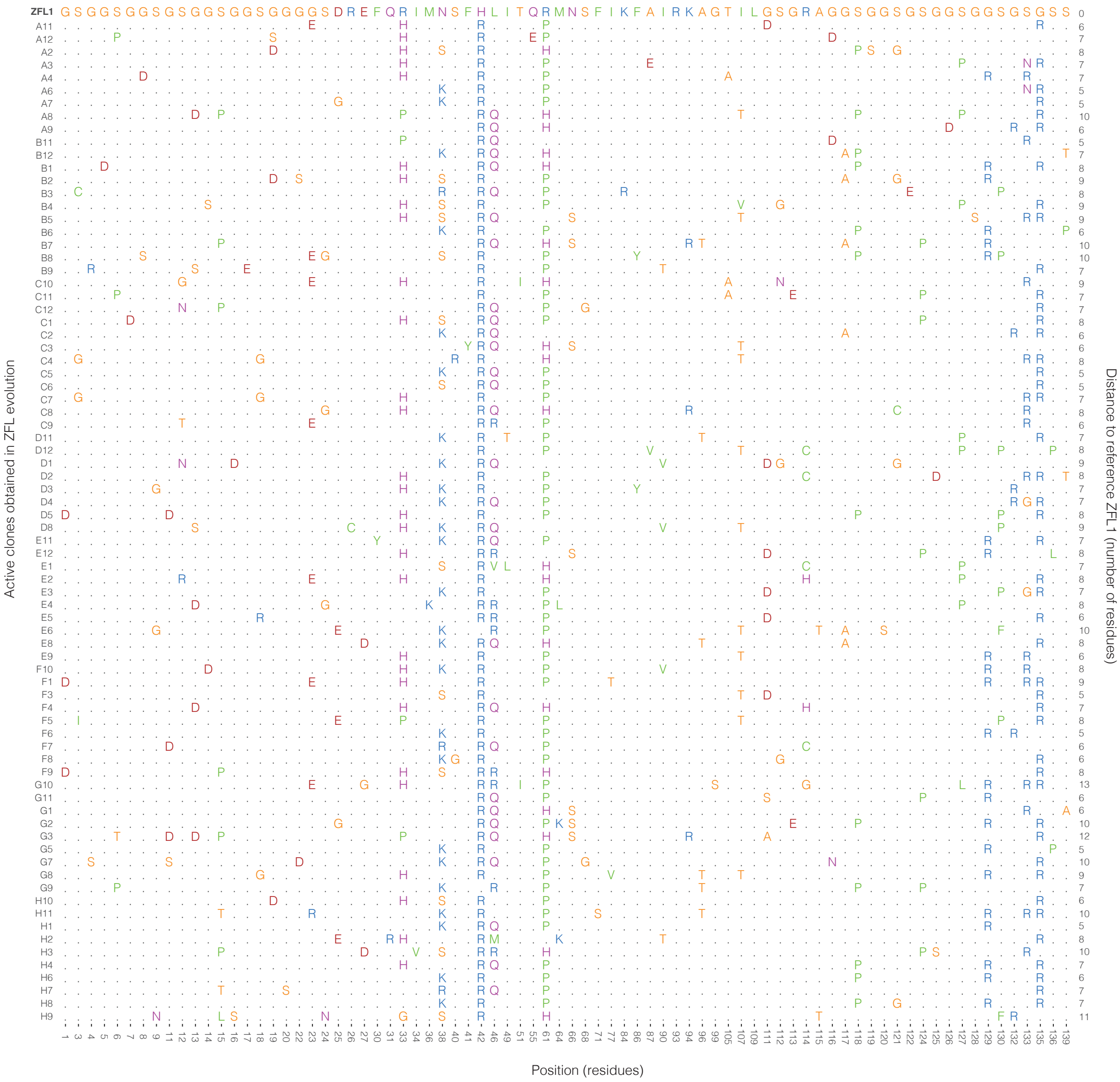

Supplementary Figure 5

Alignment of ZFL sequences obtained from the active D7L-ZFL clones after ZF-SLiDE.

One letter amino acid alignment with positions that were mutated in the sequenced clones are listed, aligned to the *in silico* designed ZFL1 that served as a starting point. Amino acids are colored according to their properties (small nonpolar (G, A, S, T) - orange, hydrophobic (C, V, I, L, P, F, Y, M, W) – green, polar (N, Q, H) - magenta, negatively charged (D, E) – red, positively charged (K, R) - blue).

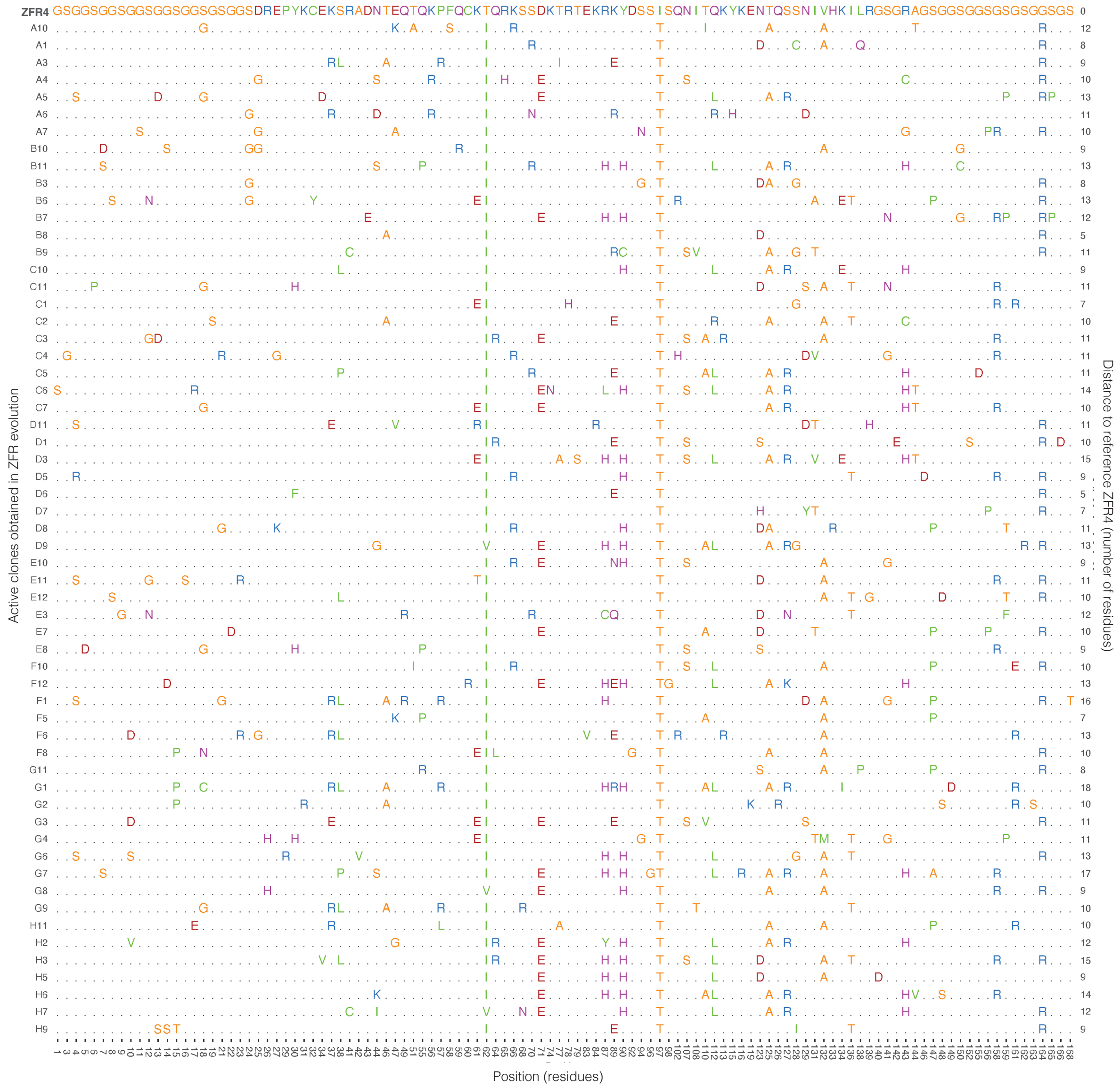

Supplementary Figure 6

Alignment of ZFR sequences obtained from the active D7R-ZFR clones after ZF-SLiDE.

One letter amino acid alignment with positions that were mutated in the sequenced clones are listed, aligned to the *in silico* designed ZFR4 that served as a starting point. Amino acids are colored according to their properties (small nonpolar (G, A, S, T) - orange, hydrophobic (C, V, I, L, P, F, Y, M, W) – green, polar (N, Q, H) - magenta, negatively charged (D, E) – red, positively charged (K, R) - blue).

Supplementary References

1. Ennifar, E. Crystal structure of a wild-type Cre recombinase-loxP synapse reveals a novel spacer conformation suggesting an alternative mechanism for DNA cleavage activation. *Nucleic Acids Res.* **31**, 5449–5460 (2003).
2. Tomasello, G., Armenia, I. & Molla, G. The Protein Imager: a full-featured online molecular viewer interface with server-side HQ-rendering capabilities. *Bioinformatics* **36**, 2909–2911 (2020).
