# Supplementary material for: Activation of recombinases at specific DNA loci by zinc-finger domain insertions
Source: Nat Biotechnol. 2024 Jan 31;42(12):1844–54. doi: 10.1038/s41587-023-02121-y (PMC11631766; doi:10.1038/s41587-023-02121-y)
Supplement: Supplementary file 2 — Reporting Summary [file 41587_2023_2121_MOESM2_ESM.pdf]

nature portfolio

Corresponding author(s): Frank Buchholz

Last updated by author(s): Dec 20, 2023

Reporting Summary

Nature Portfolio wishes to improve the reproducibility of the work that we publish. This form provides structure for consistency and transparency in reporting. For further information on Nature Portfolio policies, see our Editorial Policies and the Editorial Policy Checklist.

Statistics

For all statistical analyses, confirm that the following items are present in the figure legend, table legend, main text, or Methods section.

n/a	Confirmed	
☐	☒	The exact sample size (*n*) for each experimental group/condition, given as a discrete number and unit of measurement
☐	☒	A statement on whether measurements were taken from distinct samples or whether the same sample was measured repeatedly
☐	☒	The statistical test(s) used AND whether they are one- or two-sided<br>*Only common tests should be described solely by name; describe more complex techniques in the Methods section.*
☒	☐	A description of all covariates tested
☒	☐	A description of any assumptions or corrections, such as tests of normality and adjustment for multiple comparisons
☐	☒	A full description of the statistical parameters including central tendency (e.g. means) or other basic estimates (e.g. regression coefficient) AND variation (e.g. standard deviation) or associated estimates of uncertainty (e.g. confidence intervals)
☐	☒	For null hypothesis testing, the test statistic (e.g. *F*, *t*, *r*) with confidence intervals, effect sizes, degrees of freedom and *P* value noted<br>*Give P values as exact values whenever suitable.*
☒	☐	For Bayesian analysis, information on the choice of priors and Markov chain Monte Carlo settings
☒	☐	For hierarchical and complex designs, identification of the appropriate level for tests and full reporting of outcomes
☒	☐	Estimates of effect sizes (e.g. Cohen's *d*, Pearson's *r*), indicating how they were calculated

Our web collection on statistics for biologists contains articles on many of the points above.

Software and code

Policy information about availability of computer code

Data collection	Sequencing data was processed with exonerate v2.3.0, guppy v5.0.7, minimap2 v2.17, samtools v1.11, GNU Awk v5.1.1, bwa-mem2 aligner v2.2.1
Data analysis	Sequencing data analysis was performed using R v4.1.1 with tidyverse v1.3.1, dplyr v1.1.1, SequenceTools v0.0.1 (https://github.com/ltschmitt/SequenceTools), ggplot2 v3.4.2, Picard MarkDuplicates tool, Genrich (ENCODE blacklist (v2)), MEME-ChIP (MEME suite, v5.5.3), BEDTools v2.30.0, FIMO v5.5.3, ZF-rec_analyses v1.1 (https://github.com/ltschmitt/ZF-rec_analyses). Flow cytometry analysis was performed with FlowJo v10.9.0. The band intensities of the bands were calculated using Fiji (Version 2.0.0.-rc-65/1.52a).

For manuscripts utilizing custom algorithms or software that are central to the research but not yet described in published literature, software must be made available to editors and reviewers. We strongly encourage code deposition in a community repository (e.g. GitHub). See the Nature Portfolio guidelines for submitting code & software for further information.

Data

Policy information about availability of data

All manuscripts must include a data availability statement. This statement should provide the following information, where applicable:

- Accession codes, unique identifiers, or web links for publicly available datasets
- A description of any restrictions on data availability
- For clinical datasets or third party data, please ensure that the statement adheres to our policy

The sequence data generated in this study are deposited in the Sequence Read Archive with the accession number PRJNA1047027. Source data is provided.

Research involving human participants, their data, or biological material

Policy information about studies with human participants or human data. See also policy information about sex, gender (identity/presentation), and sexual orientation and race, ethnicity and racism.

Reporting on sex and gender	F8 iht1h-iPSCs - male donor.
Reporting on race, ethnicity, or other socially relevant groupings	N/A
Population characteristics	N/A
Recruitment	N/A
Ethics oversight	N/A

Note that full information on the approval of the study protocol must also be provided in the manuscript.

Field-specific reporting

Please select the one below that is the best fit for your research. If you are not sure, read the appropriate sections before making your selection.

☒ Life sciences ☐ Behavioural & social sciences ☐ Ecological, evolutionary & environmental sciences

For a reference copy of the document with all sections, see nature.com/documents/nr-reporting-summary-flat.pdf

Life sciences study design

All studies must disclose on these points even when the disclosure is negative.

Sample size	Sample sizes were determined based on other studies in the field of genome editing (e.g. Karpinski et. al 2016, Lansing et al. 2022).
Data exclusions	No data was excluded
Replication	Activity assays of recombinases and ZF-recombinases (PCR-based or plasmid-based) were reproduced (n=3) and successful.<br>Zinc Finger directed evolution: The protocol for evolution of recombinases is reproducible (>300 evolutions were performed in the Buchholz Lab). The here described directed evolution for the ZFL and ZFR was not replicated.<br>Recombination assays in human cells were reproduced (n=3) and successful. All attempts at experiments replication were successful.
Randomization	No randomization was performed, because picking individual colonies from bacterial plates is random.
Blinding	Bacterial and mammalian cell experiment were performed under the same conditions. No blinding was used in this study.

Reporting for specific materials, systems and methods

We require information from authors about some types of materials, experimental systems and methods used in many studies. Here, indicate whether each material, system or method listed is relevant to your study. If you are not sure if a list item applies to your research, read the appropriate section before selecting a response.

Materials & experimental systems

n/a	Involved in the study
☐	☒ Antibodies
☐	☒ Eukaryotic cell lines
☒	☐ Palaeontology and archaeology
☒	☐ Animals and other organisms
☒	☐ Clinical data
☒	☐ Dual use research of concern
☒	☐ Plants

Methods

n/a	Involved in the study
☐	☒ ChIP-seq
☐	☒ Flow cytometry
☒	☐ MRI-based neuroimaging

Antibodies

Antibodies used: goat GFP-antibody (MPI-CBG antibody facility)

Validation: Chakraborty D, Paszkowski-Rogacz M, Berger N, Ding L, Mircetic J, Fu J, Iesmantavicius V, Choudhary C, Anastassiadis K, Stewart AF, Buchholz F. lncRNA Panct1 Maintains Mouse Embryonic Stem Cell Identity by Regulating TOBF1 Recruitment to Oct-Sox Sequences in Early G1. Cell Rep. 2017 Dec 12;21(11):3012-3021. doi: 10.1016/j.celrep.2017.11.045. PMID: 29241531.

Eukaryotic cell lines

Policy information about cell lines and Sex and Gender in Research

Cell line source(s): HEK293T - ATCC, human iPSCs - Stem Cell Engineering Core Facility of the CMCB Technology Platform at TU Dresden

Authentication: HEK293T cells were not authenticated. The human iPSCs lines were authenticated (tested for pluripotency (FACS- based) and contamination of Mycoplasma) by the Stem Cell Engineering Core Facility of the CMCB Technology Platform at TU Dresden.

Mycoplasma contamination: HEK293T cells and human iPSCs were tested negative for Mycoplasma

Commonly misidentified lines (See ICLAC register): No commonly misidentified cell lines were used in this study

Plants

Seed stocks: *Report on the source of all seed stocks or other plant material used. If applicable, state the seed stock centre and catalogue number. If plant specimens were collected from the field, describe the collection location, date and sampling procedures.*

Novel plant genotypes: *Describe the methods by which all novel plant genotypes were produced. This includes those generated by transgenic approaches, gene editing, chemical/radiation-based mutagenesis and hybridization. For transgenic lines, describe the transformation method, the number of independent lines analyzed and the generation upon which experiments were performed. For gene-edited lines, describe the editor used, the endogenous sequence targeted for editing, the targeting guide RNA sequence (if applicable) and how the editor was applied.*

Authentication: *Describe any authentication procedures for each seed stock used or novel genotype generated. Describe any experiments used to assess the effect of a mutation and, where applicable, how potential secondary effects (e.g. second site T-DNA insertions, mosiacism, off-target gene editing) were examined.*

ChIP-seq

Data deposition

☒ Confirm that both raw and final processed data have been deposited in a public database such as GEO.

☒ Confirm that you have deposited or provided access to graph files (e.g. BED files) for the called peaks.

Data access links
May remain private before publication.: https://www.ncbi.nlm.nih.gov/geo/query/acc.cgi?acc=GSE243676

Files in database submission: Sequencing reads from the D7ZF and EGFP ChIP-Seq samples (fastq format) and a table with putative binding sites of D7ZF (narrowPeak format)

Genome browser session (e.g. UCSC): No longer applicable.

Methodology

Replicates: No replicates

Sequencing depth	Approximately 40 million uniquely mapped read pairs, with the average fragment length of 294 bp.
Antibodies	goat anti-GFP (MPI-CBG antibody facility)
Peak calling parameters	Minimum MAPQ to keep an alignment: 30, secondary alignments are kept if their AS score is at least the primary alignment AS less 5.
Data quality	25 reported peaks with the maximum p-value of 0.01 and the minimum AUC of 200.
Software	Sequence alignment by bwa-mem2, using GRCh38.p13 as the reference genome. PCR and optical duplicate removal by Picard MarkDuplicates, followed by peak calling by Genrich, using the ENCODE blacklist (v2) for filtering out problematic regions.

Flow Cytometry

Plots

Confirm that:

☒ The axis labels state the marker and fluorochrome used (e.g. CD4-FITC).

☒ The axis scales are clearly visible. Include numbers along axes only for bottom left plot of group (a 'group' is an analysis of identical markers).

☒ All plots are contour plots with outliers or pseudocolor plots.

☒ A numerical value for number of cells or percentage (with statistics) is provided.

Methodology

Sample preparation	HEK293T cells were washed with PBS, followed by dissociation with Trypsin for 3 min. The detached cells were resuspended in DMEM with 10% FBS for analysis.
Instrument	MACSQuant VYB Flow Cytometer
Software	FlowJo v10.6.2
Cell population abundance	Abundance of relevant HEK293T cells within the post-sort fraction: GFP+ 8-16%; EGFP+mCherry+ 9-66% or GFP+BFP+ 14-22%. The purity was not further determined since all cells derived from a culture system with only one type.
Gating strategy	HEK293T cells gating strategy:<br>live cells: FSC-A 100-600 and SSC-A 100-650;<br>Single cells: starting population - live cells, FSC-A 50-850 and FSC-H 10-500;<br>Transfected cells (GFP+): starting population - single cells, GFP-FITC-A > 10^0; or (for GFP+BFP+): starting population - single cells, GFP-FITC-A > 10^-0.1 and CFP_VioBlue >10^-0.3.<br>Recombined cells (GFP+mCherry+): starting population - transfected cells, Tx-Red-A > 10^0.

☒ Tick this box to confirm that a figure exemplifying the gating strategy is provided in the Supplementary Information.
