# Supplementary material for: Activation of recombinases at specific DNA loci by zinc-finger domain insertions
Source: Nat Biotechnol. 2024 Jan 31;42(12):1844–54. doi: 10.1038/s41587-023-02121-y (PMC11631766; doi:10.1038/s41587-023-02121-y)

Figure 1f and Figure 1g source data

Replicate 1

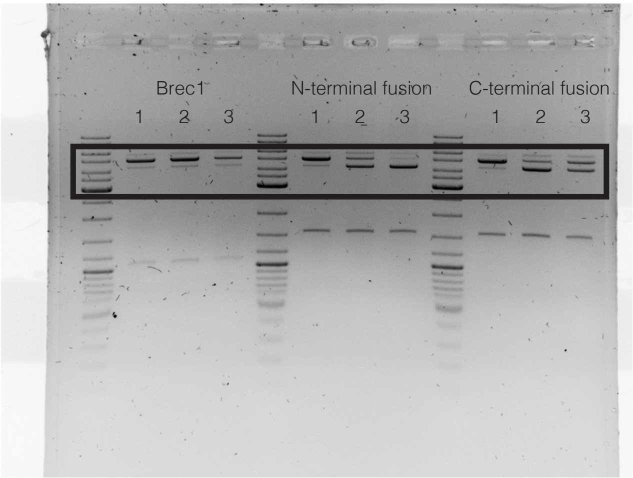

Replicate 2

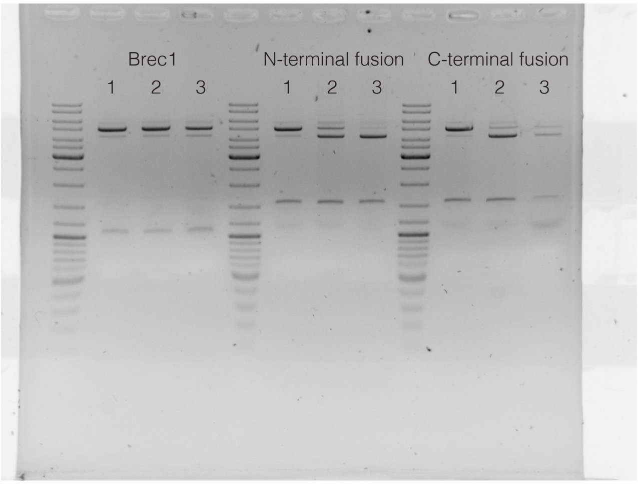

Replicate 3 (samples were on three different gels)

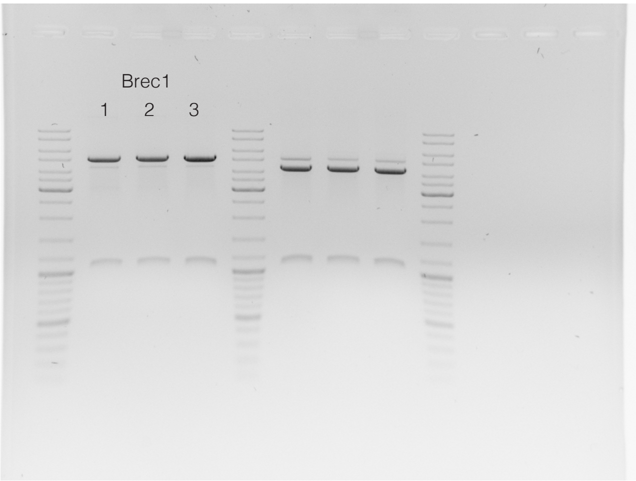

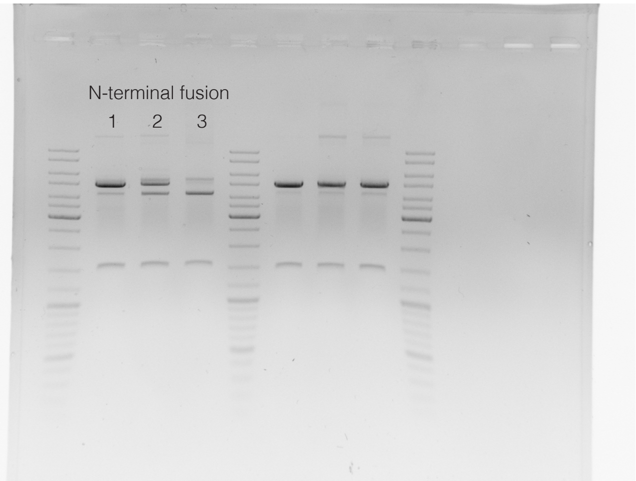

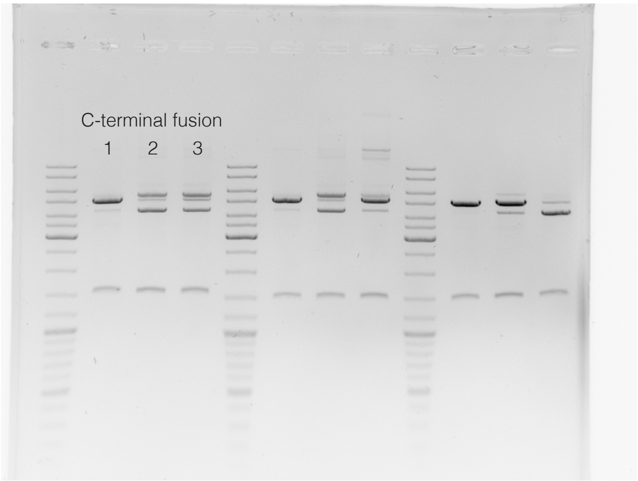

Agarose gel pictures of the replicates used for the experiment shown in Figure 1f and Figure 1g.
The black frame indicates the fragment of the gel that is shown in the Figure 1f.
TS = target site. TS1: loxBTR, TS2: loxBTR-5-zif (A), TS3: loxBTR-5-zif (B).

Figure 2e and Figure 2f source data

Replicate 1

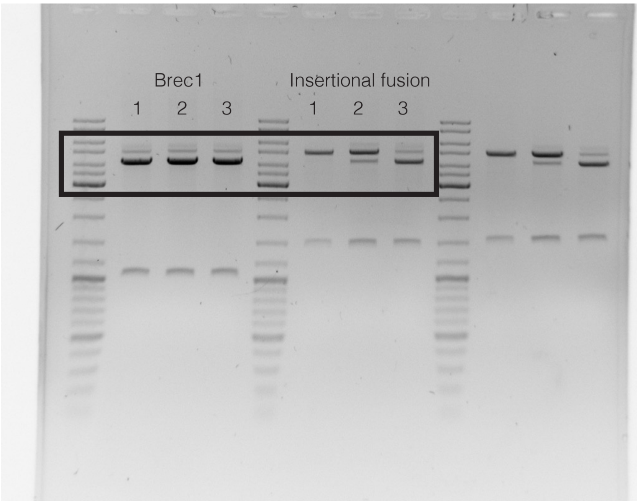

Replicate 2

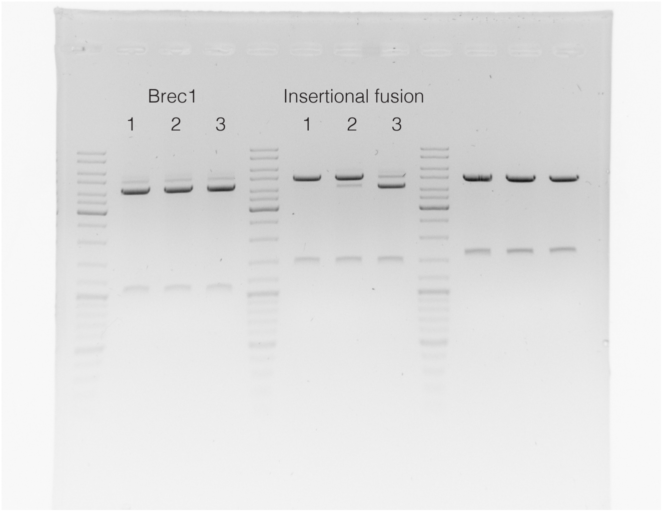

Replicate 3

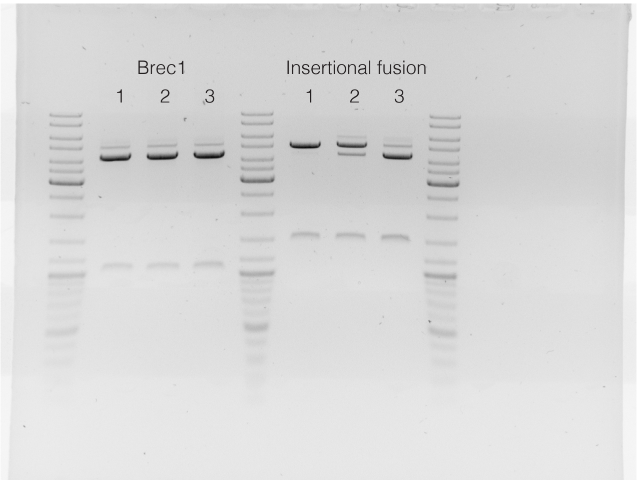

Agarose gel pictures of the replicates used for the experiment shown in Figure 2e and Figure 2f.
The black frame indicates the fragment of the gel that is shown in the Figure 2e.
TS = target site. TS1: loxBTR, TS2: loxBTR-5-zif (A), TS3: loxBTR-5-zif (B).

Figure 3b source data

Replicate 1

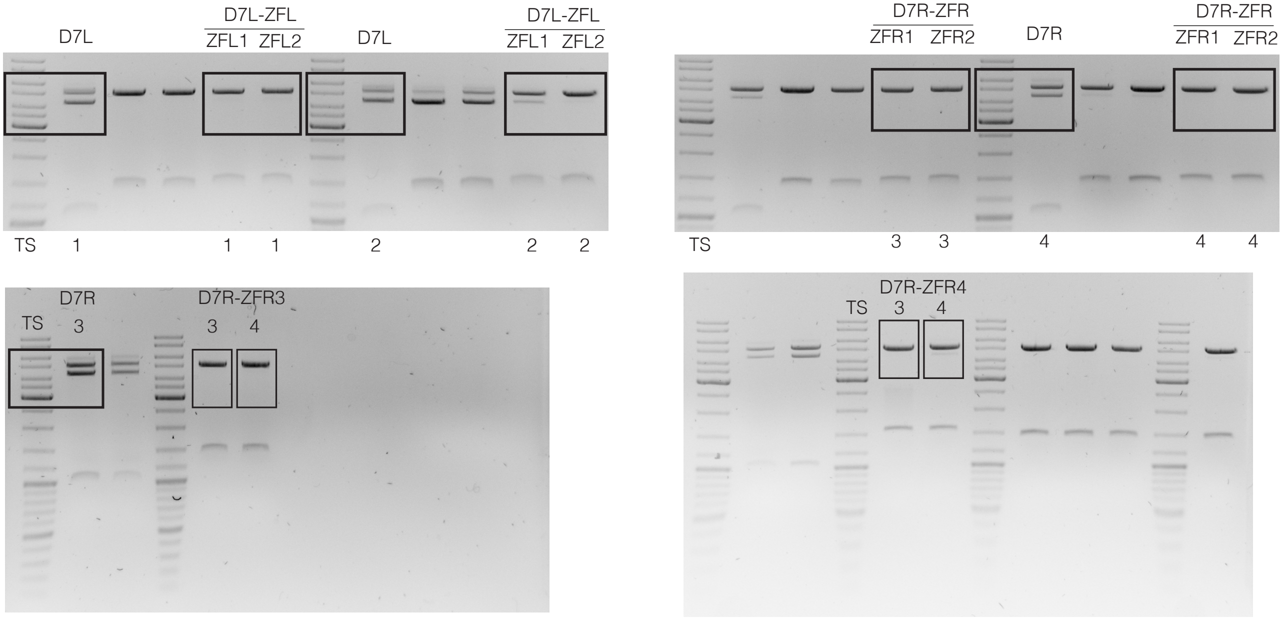

Replicate 2

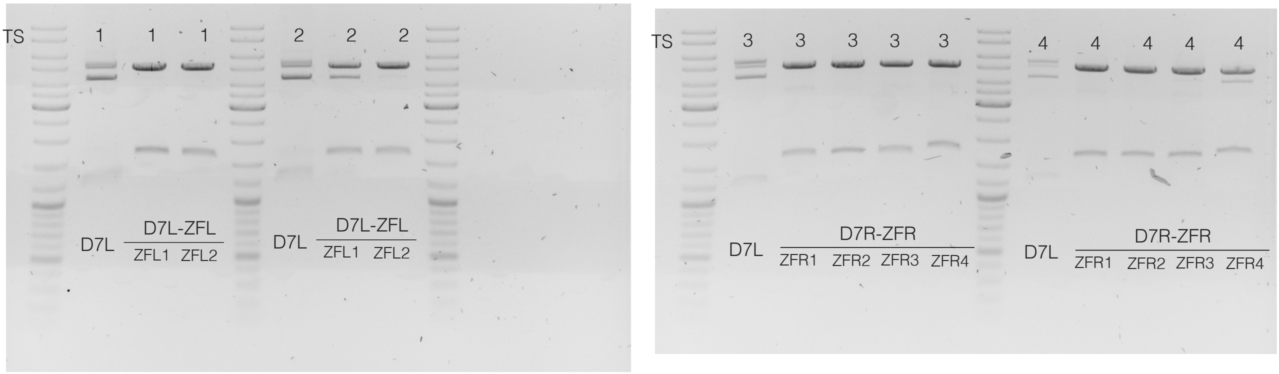

Replicate 3

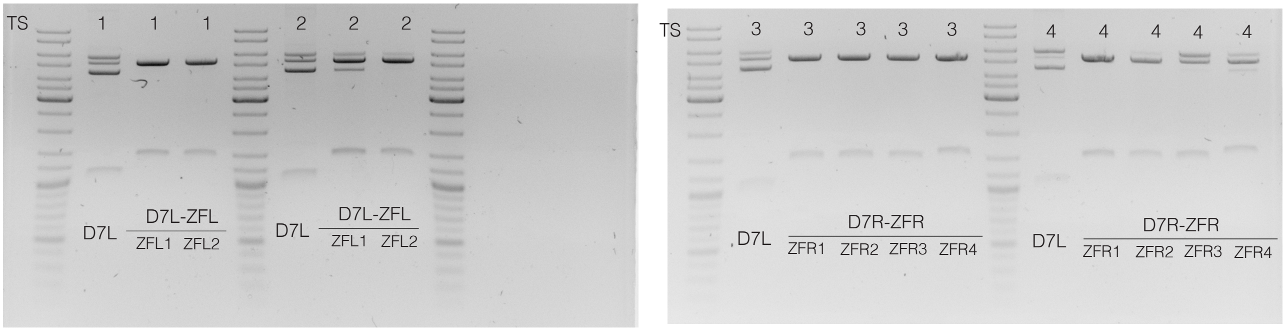

Agarose gel pictures of the replicates used for the experiment shown in Figure 3b. The black frames indicate the fragments of the gels that are shown in the Figure 3b. TS = target site. TS1: loxF8L, TS2: loxF8L-flank, TS3: loxF8R, TS4: loxF8R-flank.

Figure 4b source data

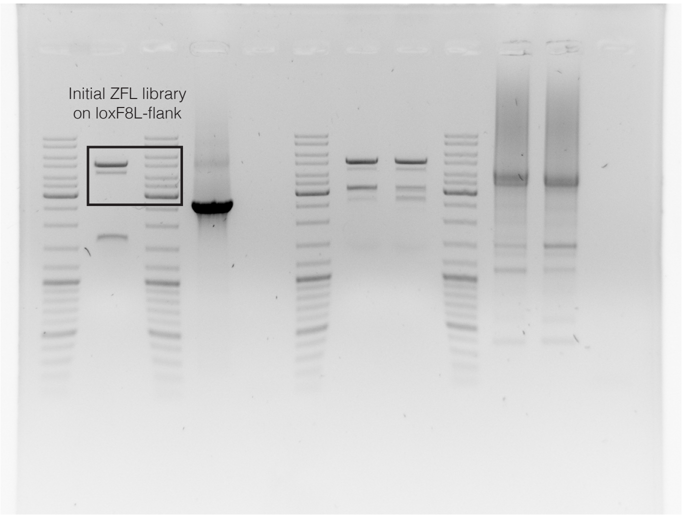

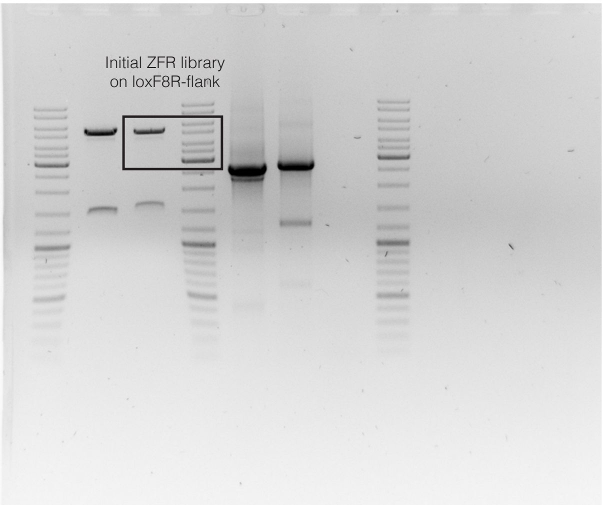

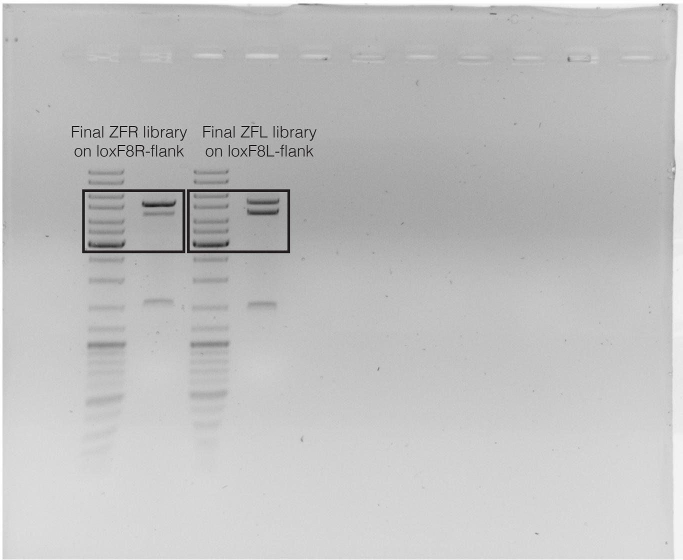

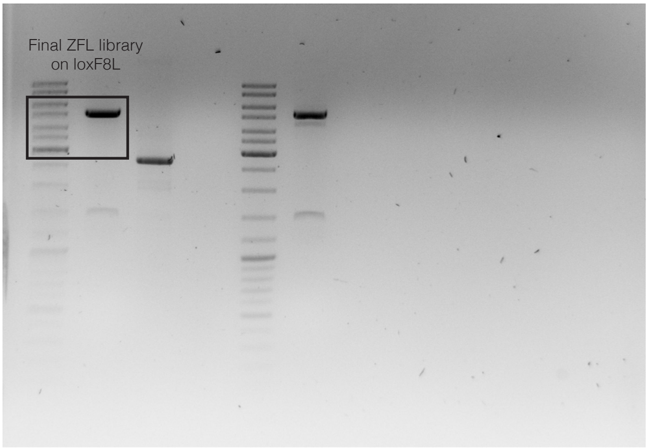

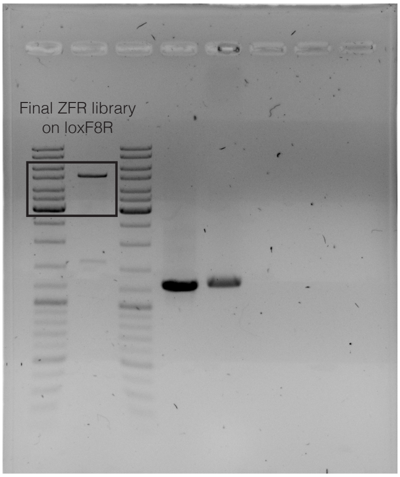

Agarose gel pictures of the recombination assay of the D7L and D7R recombinases fused with ZFL and ZFR libraries in the initial and final cycles of ZF evolution (n=1). The black frames indicate the fragments of the gels that are shown in the Figure 4b.

Figure 5b and Figure 5c source data

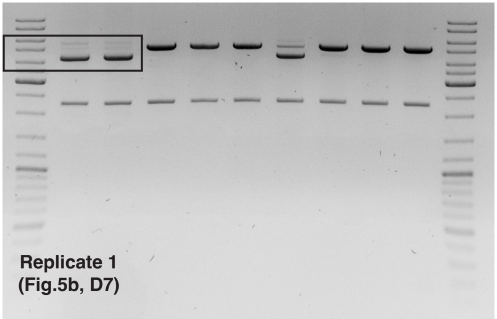

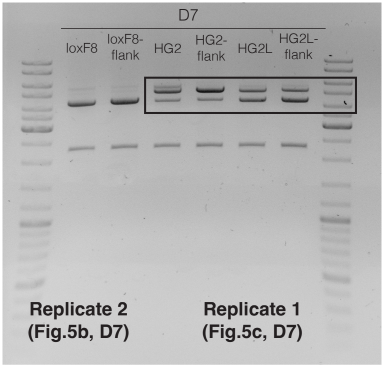

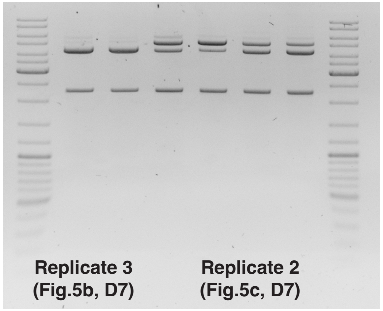

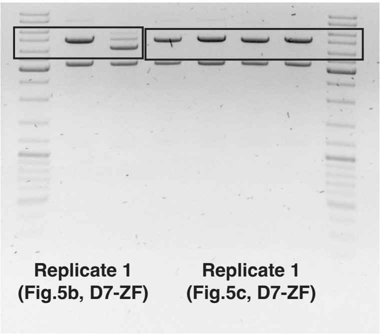

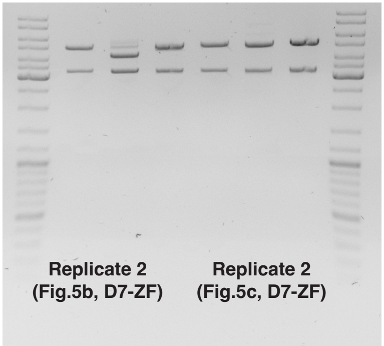

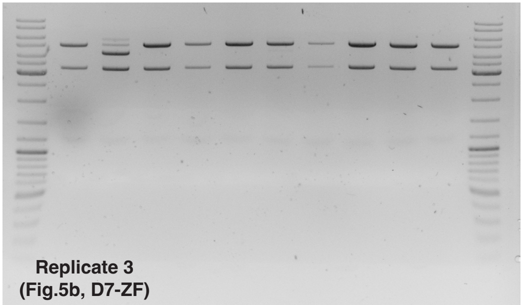

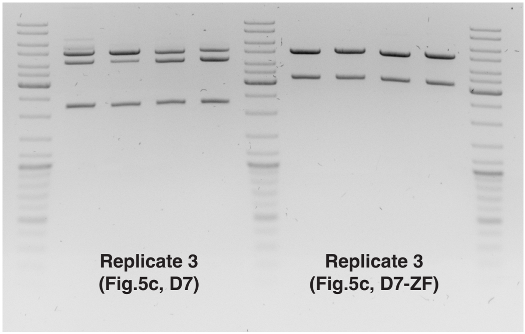

Agarose gel pictures of the replicates used for the experiments shown in the Figure 5b and Figure 5c. The black frames indicate the fragments of the gels that are shown in the Figure 5b and Figure 5c.

Figure 5d source data

Replicate 1

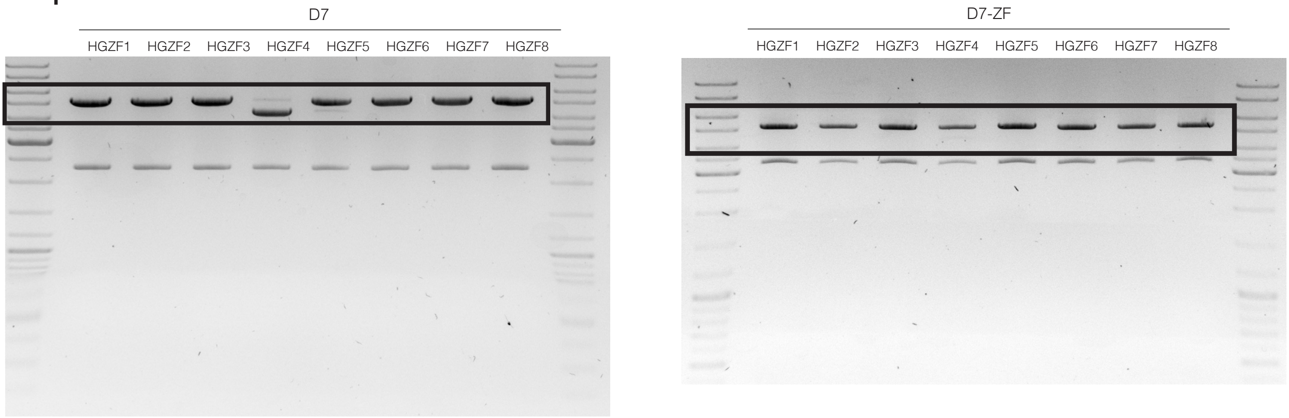

Replicate 2

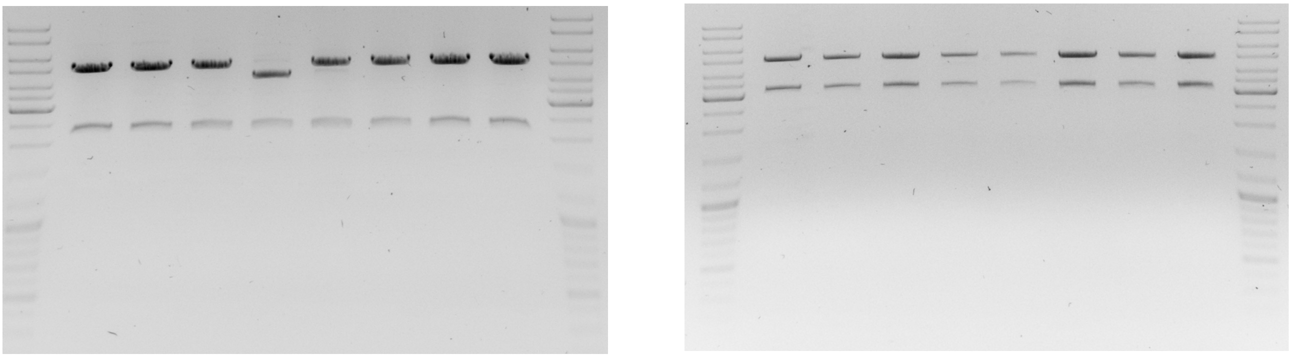

Replicate 3

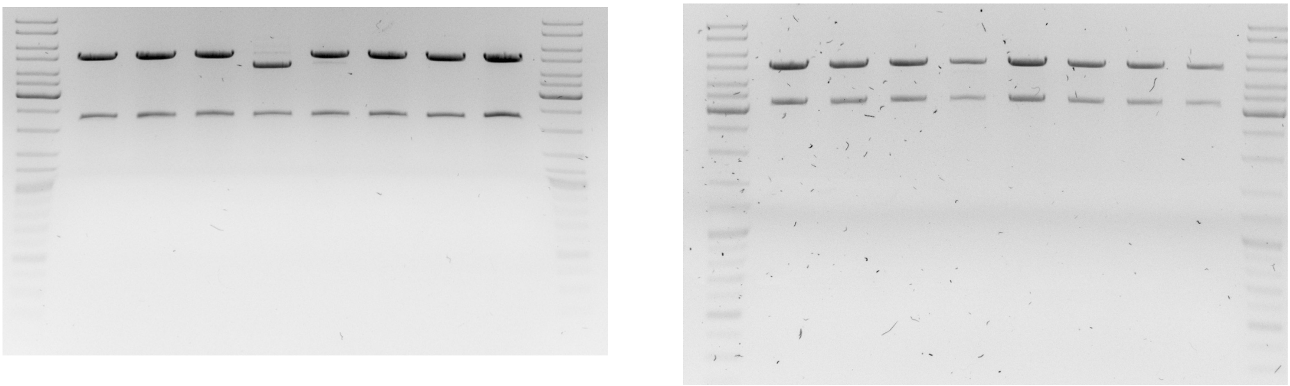

Agarose gel pictures of the replicates used for the experiment shown in Figure 5d. The black frames indicate the fragments of the gels that are shown in the Figure 5d.

Figure 5f source data

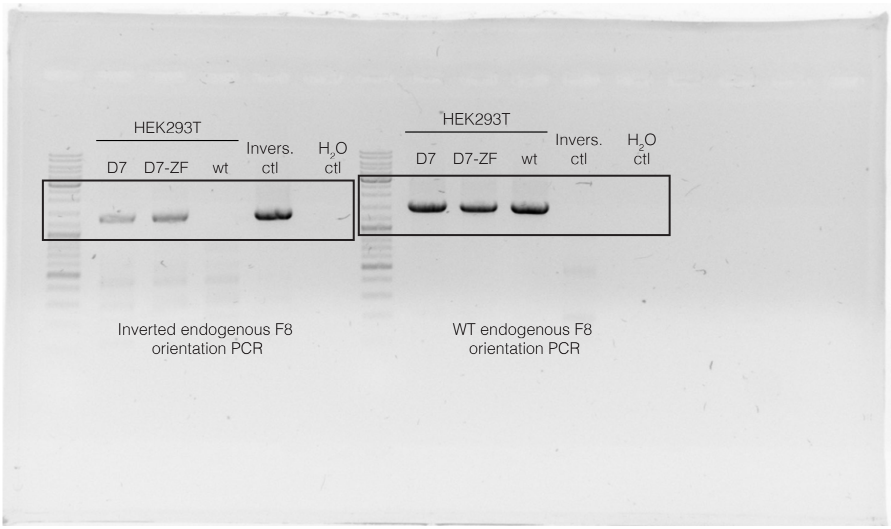

Figure 6d source data

Replicate 1

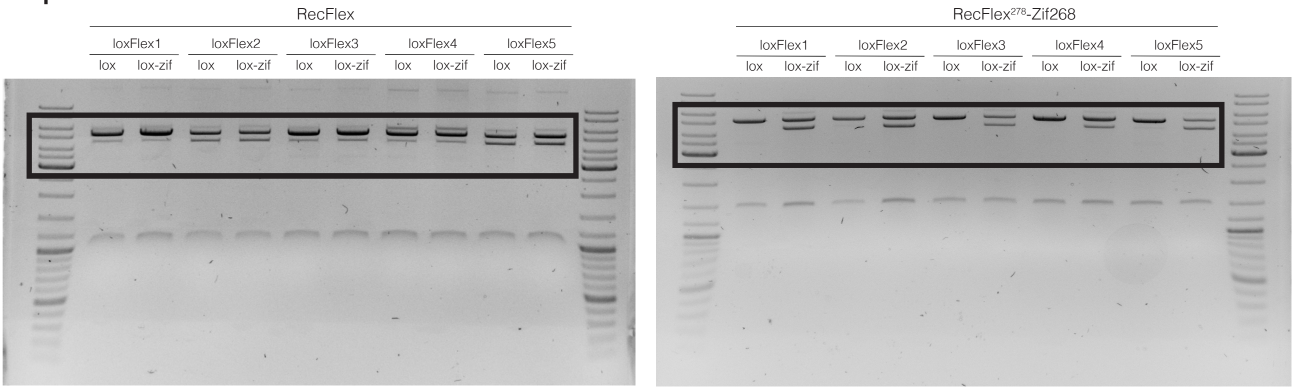

Replicate 2

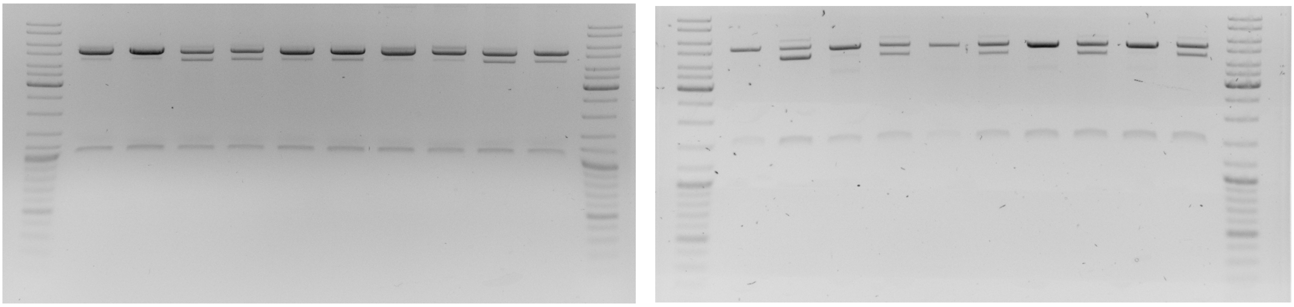

Replicate 3

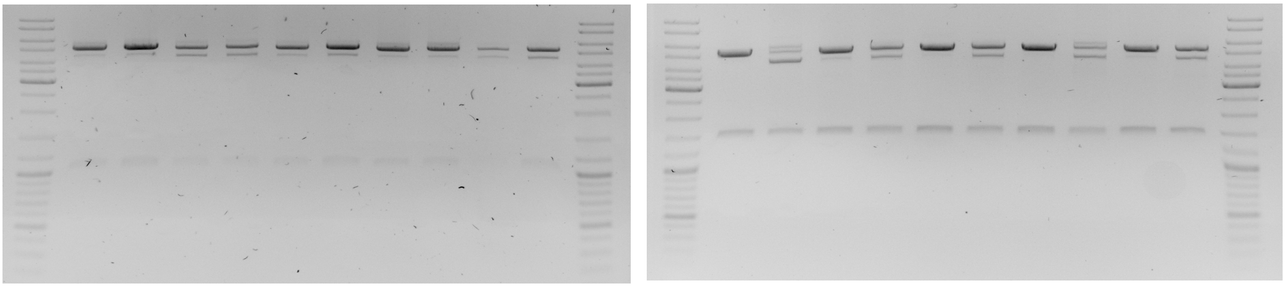

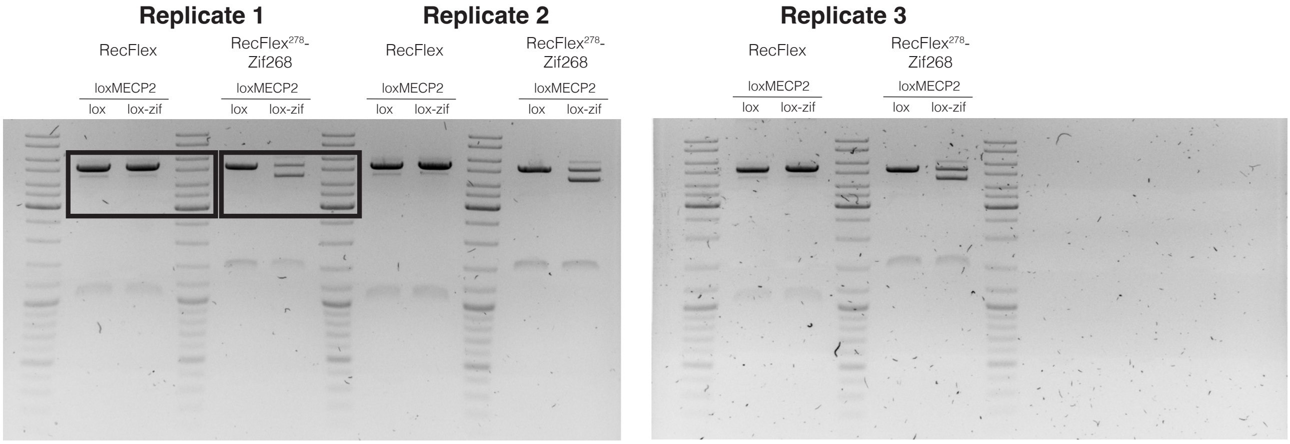

Agarose gel pictures of the replicates used for the experiment shown in Figure 6d. The black frames indicate the fragments of the gels that are shown in the Figure 6d.

Extended Data Fig 6e source data

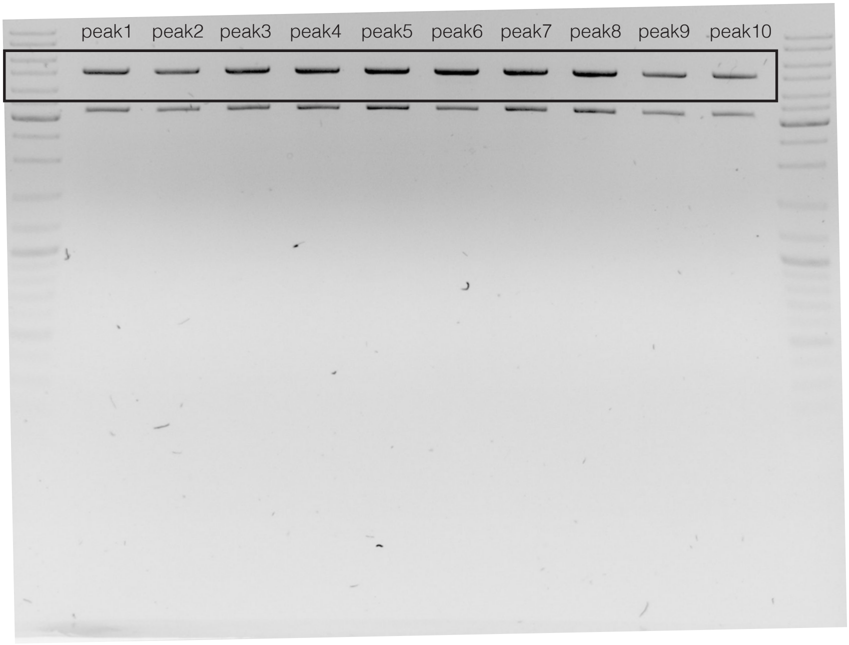

Extended Data Figure 8 source data

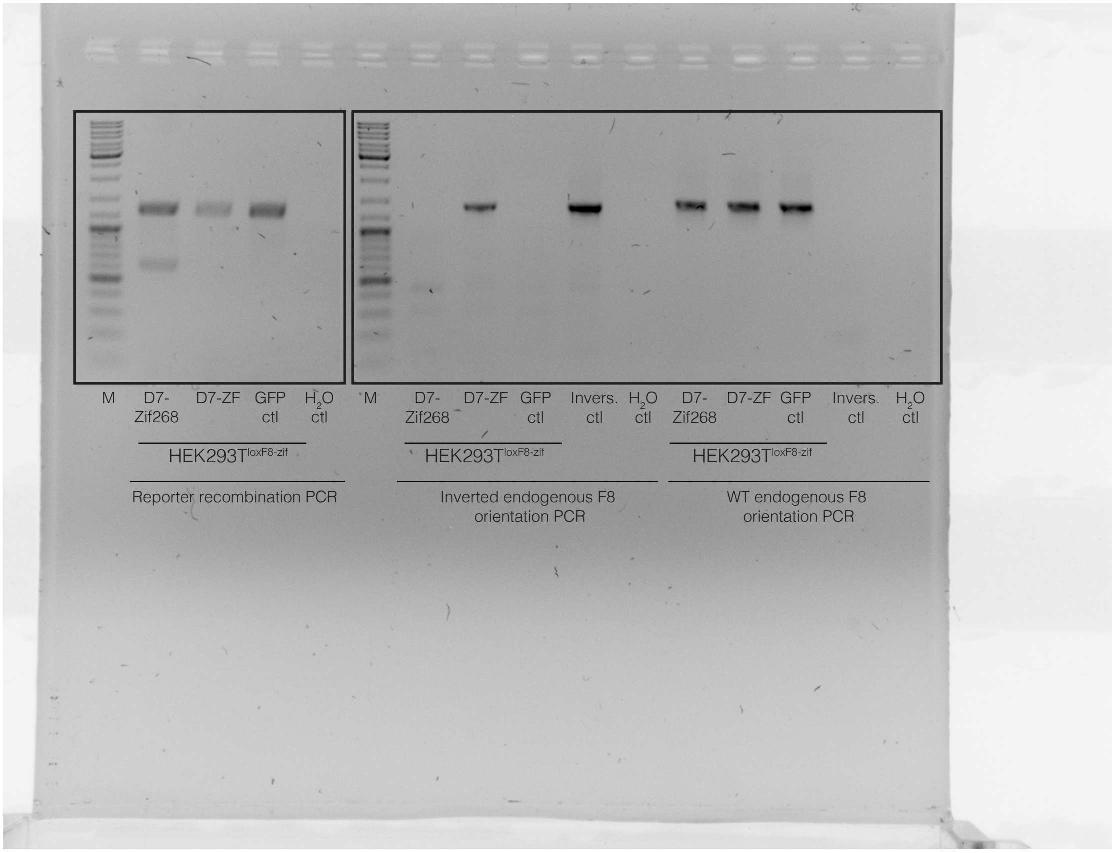

Extended data Figure 9a source data

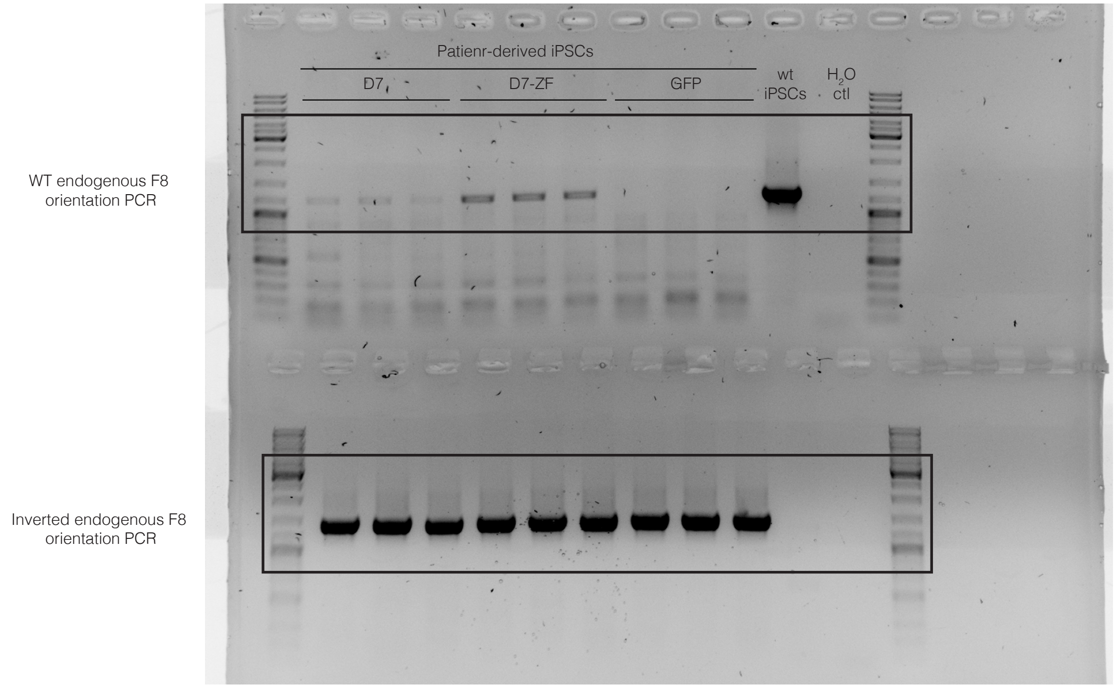

Supplement: Supplementary file 4 — Unprocessed gels of Figs. 1f,g, 2e,f, 3b, 4b, 5b–d,f and 6d and Extended Data Figs. 6e, 8 and 9a. [file 41587_2023_2121_MOESM4_ESM.pdf]
